# STARD4 suppresses tumorigenesis and attenuates enzalutamide resistance via lipid metabolic reprogramming and AR stabilization in prostate cancer

**DOI:** 10.1186/s13046-025-03600-7

**Published:** 2025-12-02

**Authors:** Yi Zhang, Xi Wang, Jiuyi Wang, Ke Ma, Lei Jia, Bo Liu, Xianglin Yuan, Qiang Li, Qinzhang Wang, Qinyu Li, Kai Zeng

**Affiliations:** 1 Department of Urology, the First Affiliated Hospital of Shihezi University, Shihezi, Xinjiang China; 2 Department of Oncology, Tongji Hospital, Tongji Medical College, Huazhong University of Science and Technology, Wuhan, Hubei China

**Keywords:** Prostate cancer, STARD4, Lipid metabolism, Androgen receptor, Enzalutamide resistance

## Abstract

**Background:**

Prostate cancer (PCa) is a globally prevalent malignancy in males and is imposing an increasing epidemiological burden. The androgen receptor (AR) signalling axis is fundamentally implicated in PCa tumorigenesis and disease progression. Although androgen deprivation therapy (ADT) elicits transient therapeutic responses in the majority of cases, progression to castration-resistant prostate cancer (CRPC) remains an almost universal clinical trajectory. Dysregulated lipid homeostasis, manifesting as intracellular lipid deposition, has been mechanistically linked to CRPC pathogenesis and therapeutic failure under enzalutamide regimens. However, effective strategies to mitigate lipid accumulation in PCa remain elusive.

**Methods:**

STARD4, a key gene involved in lipid metabolism, was identified as functionally significant in PCa through integrated bioinformatics analysis of public databases. RT‒qPCR, western blot analysis, and IHC staining were performed to evaluate STARD4 expression, while Kaplan–Meier survival analysis, Gleason score, and tumor stage were performed to assess its clinical significance in PCa. The biological functions of STARD4 and its contribution to enzalutamide resistance were elucidated through in vitro and in vivo experiments. The effect of STARD4 on abnormal lipid accumulation in PCa cells was evaluated by Oil Red O (ORO) staining, while its impact on endoplasmic reticulum (ER) stress was assessed through ER-tracking imaging and transmission electron microscopy (TEM). Mechanistic exploration involves a combination of techniques, including RNA-seq analysis, Gene ontology analysis, coimmunoprecipitation (Co-IP), and GST pull-down assay, to analyse the interactions and potential mechanisms involving STARD4, AR, and E3 ubiquitin ligase UBE4B.

**Results:**

In this study, we observed that STARD4 expression was markedly reduced in PCa tissues and was correlated with an adverse prognosis. STARD4 overexpression inhibited PCa cell proliferation, migration, and lipid accumulation while promoting apoptosis through ER stress. Mechanistically, STARD4 enhanced the interaction between UBE4B and AR, facilitating AR ubiquitination and degradation and thus suppressing AR signalling. Additionally, the upregulation of STARD4 expression enhanced sensitivity to enzalutamide in resistant cells by diminishing lipid accumulation and inhibiting the AR signalling pathway. In summary, STARD4 functions as a tumour suppressor in PCa by regulating cholesterol metabolism and modulating AR signalling.

**Conclusions:**

Our findings identify STARD4 as a promising therapeutic target for reversing enzalutamide resistance in PCa while also providing novel insights for future research on lipid metabolism within the tumour microenvironment.

**Graphical abstract:**

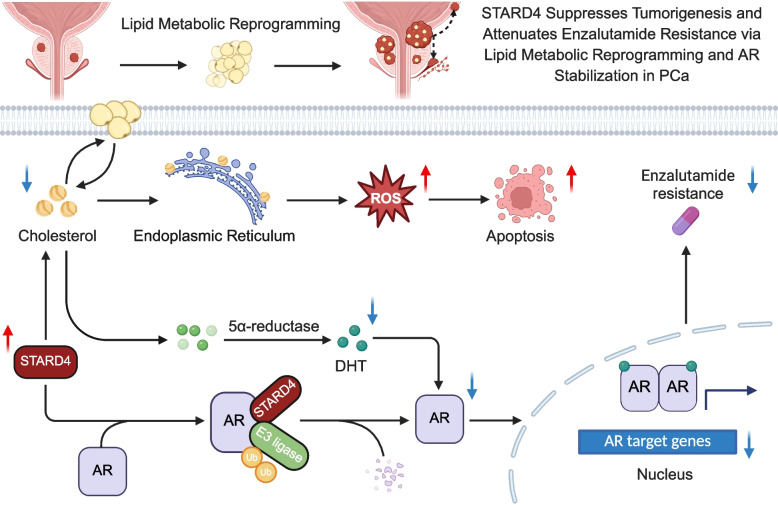

**Supplementary Information:**

The online version contains supplementary material available at 10.1186/s13046-025-03600-7.

## Introduction

PCa ranks as one of the most prevalent malignancies among men [1, 2]. The androgen signalling pathway plays a pivotal role in the initiation and progression of PCa [3]. Consequently, while traditional treatments such as surgery and radiation can enhance patient outcomes, AR-targeted therapies remain the cornerstone of advanced PCa treatment because of their specificity and efficacy [4, 5]. However, the initial sensitivity of most patients to ADT wanes to the nearly inevitable development of CRPC, underscoring the intricate nature of the androgen signalling pathway in PCa [6, 7]. Therefore, further research is needed to elucidate the mechanisms underlying resistance to AR-targeted therapies in PCa and to propose innovative treatment strategies to address this formidable clinical challenge.

Elevated lipid metabolism has long been recognized to sustain the proliferation of tumour cells, including liver cancer, breast cancer, and PCa cells [8–10]. Lipid accumulation resulting from aberrant lipid metabolism in PCa is considered a crucial factor in the development of CRPC and resistance to enzalutamide [11, 12]. Emerging evidence has revealed that AR transcriptionally regulates lipid anabolism through the coordinated upregulation of the expression of key enzymes, including long-chain acyl-CoA synthetase 3 (ACSL3), membrane-bound O-acyltransferase domain-containing 2 (MBOAT2), and the elongation of very long-chain fatty acids 5/7 (ELOVL5/7) [13–15]. Similarly, the AR-dependent activation of sterol regulatory element-binding protein 2 (SREBP2) drives de novo cholesterol biosynthesis, further amplifying lipidogenic reprogramming in PCa [16]. Accordingly, cholesterol, a precursor of androgen synthesis, expedites androgen production and sustains AR signalling activation [17–19]. The bidirectional interaction between SREBP and androgens forms a self-amplifying loop that promotes abnormal lipid accumulation and AR activation, a mechanism known to underlie CRPC and enzalutamide resistance [3, 11, 12]. Therefore, identifying effective strategies to alleviate abnormal lipid accumulation in tumour cells may represent a promising therapeutic avenue for CRPC.

Members of the steroidogenic acute regulatory protein-associated lipid transport (START) domain family are involved in nonvesicular lipid transport, metabolism, and tumour suppression through their ability to bind a variety of ligands, including cholesterol, phospholipids, and sphingolipids [20]. STARDs, characterized by the presence of the START domain, serve as a common lipid-binding domain. Notably, STARD4 functions as an efficient intracellular sterol transporter, facilitating cholesterol transport between the plasma membrane and the ER [20, 21]. Research has highlighted the association of STARD4 with the development of multiple malignancies, such as hepatocellular carcinoma, breast cancer, head and neck squamous cell carcinoma, and skin cutaneous melanoma [22–25]. In this study, we found that STARD4 mitigates PCa progression by mediating cholesterol metabolism and ER function. Furthermore, we discovered that STARD4 not only inhibits androgen synthesis but also promotes the ubiquitination and degradation of AR and that STARD4 overexpression markedly curtails the growth of enzalutamide-resistant PCa.

## Materials and methods

### Cell lines and tissue samples

Human PCa cell lines (DU145, PC3, C4-2, and LNCaP) and HEK293T cells were obtained from American Type Culture Collection (ATCC; Manassas, USA) and cultured in accordance with ATCC guidelines. The 22Rv1 cell line was procured from ProCell (Wuhan, China) and maintained in RPMI-1640 medium (Boster, Wuhan, China). The immortalized human prostatic epithelial cell line RWPE-1 was also obtained from ProCell and cultured in K-SMF medium (Gibco, USA). To develop a C4-2-EnzR cell line, C4-2 cells were initially treated with 4 μM enzalutamide (MCE; Shanghai, China), and the concentration was progressively increased to 40 μM over a four-month period. Thereafter, C4-2-EnzR cells were propagated in RPMI-1640 medium supplemented with 20 μM enzalutamide (MCE; Shanghai, China). All the cell lines were subjected to STR profiling verification and cultured under standardized conditions (37 °C, 5% CO₂, and 95% humidity).

Tumour tissues were collected from 77 PCa patients who underwent radical prostatectomy at the First Affiliated Hospital of Shihezi University from September 2021 to May 2023. This research was authorized by the Ethics Committee of the First Affiliated Hospital of Shihezi University (KJ2024-042–01). Written informed consent was obtained from the patients for the use of tissue samples in scientific research.

### Plasmids and cell transfection

Short hairpin RNA and nontarget shRNAs were designed and synthesized by Genomeditech (Shanghai, China): sh-STARD4 #1: 5′-TCCTATACTGTGGGCTATAAATTCAAGAGAGATTTATAGCCCACAGTATAGGATTTTT-3′; sh-STARD4 #2: 5′-ACAAAGCCCAAGGTGTTATAGTTCAAGAGACTATAACACCTTGGGCTTTGTTTTTTT-3′; and sh-STARD4 #3: 5′-CCGGTCCTATACTGTGGGCTATAAACTCGAGTTTATAGCCCCACAGTATAGGATTTTTG-3′. Flag-tagged AR (Gene ID: 367; vector: pGMLV), Flag-tagged UBE4B (Gene ID: 10,277; vector: pGMLV), Flag-tagged FANCG (Gene ID: 2189; vector: pGMLV), and STARD4 overexpression plasmids were also sourced from Genomeditech. His-tagged STARD4 (Gene ID: 134,429; vector: pGMLV) and GST-tagged AR (Gene ID: 367; vector: pGEX-4 T-1) were procured from Tsingke Biotechnology (Beijing, China). Additionally, HA-tagged STARD4 (Gene ID: 134,429; vector: pCMV), and AR truncation domain (Flag-NTD, Flag-NTD-DBD, Flag-DBD, Flag-DBD-LBD, and Flag-LBD) plasmids were designed and synthesized by Miaoling Biotechnology (Wuhan, China). Transfection efficiency was rigorously assessed via RT‒qPCR and western blotting.

### CCK-8, EdU, and colony formation assays

For proliferation assays, cells were seeded in 96-well plates at an initial density of 3 × 10^3^ cells/well. Viability was quantified spectrophotometrically (OD450 nm) after incubating cells with CCK-8 reagent (Yeasen Biotechnology) per the manufacturer’s guidelines. EdU incorporation assays (RiboBio) were performed by incubating cells (5 × 10^4^/well in 12-well plates) with 50 μM EdU for 2 h, followed by fixation (4% PFA, 20 min), permeabilization (0.5% Triton X-100), and dual staining with Hoechst and Apollo. EdU⁺ cells were enumerated across five microscopic fields (Olympus IX71; 200 × magnification). Clonogenic potential was assessed after fixing (methanol) and staining (crystal violet; 0.1%, 30 min) 14-day cultures in 6-well plates, with colonies counted using ImageJ software.

### Transwell assays

The migration potential of cells was evaluated using a transwell migration assay. In brief, cells (3 × 10^4^) were seeded in the upper chamber of transwell inserts in serum-free medium; the upper chambers were placed in each well of a 24-well culture plate and immersed in 500 μl of medium supplemented with 10% FBS. After incubation for 24 h, the migrated cells located on the lower side of the membrane were immobilized with a 4% paraformaldehyde solution. After staining the cells with 0.1% crystal violet solution, cells in five randomly selected fields were subsequently enumerated using a microscope.

### Flow cytometry analysis of cell apoptosis

Flow cytometry was performed to determine the percentage of apoptotic cells. Following plasmid transfection or drug treatment, the cells were harvested and incubated with PI and Annexin V (Yeasen Biotechnology, Shanghai, China) in the dark at room temperature for 15 min. Flow cytometry analysis was performed using a CytoFlex cytometer (Beckman Coulter, USA), and the data were analysed using FlowJo V10 software.

### RT‒qPCR and RNA sequencing (RNA‒seq)

Total RNA was isolated using TRIzol™ (Servicebio Technology) and quantified spectrophotometrically. Reverse transcription was performed with the Hifair II cDNA Synthesis System (Yeasen Biotechnology). Quantitative PCR amplification was executed on a StepOnePlus™ Real-Time PCR System (Applied Biosystems) with SYBR Green chemistry. The primer sequences are detailed in Supplementary Table 1. To elucidate the transcriptomic alterations in response to STARD4 overexpression, sequencing libraries were prepared from total RNA isolated from both the control and STARD4-overexpressing groups. These libraries were then subjected to next-generation sequencing to delineate the transcriptomic landscape.

### Western blotting and Co-IP

Western blotting was carried out using standard protocols as previously described [26]. The specifications of all primary and secondary antibodies are listed in Supplementary Table 2. For Co-IP, the procedure outlined by the manufacturer (Biolinkedin, Shanghai, China) was followed. Briefly, treated cells were collected and lysed on ice for 30 min using a lysis buffer containing 1% protease and phosphatase inhibitors (Boster, Wuhan, China). The resulting lysates were subjected to overnight incubation at 4 °C with rotation in the presence of target-specific antibodies to allow immune complex formation. Subsequently, prechilled magnetic beads were introduced and incubated with the complexes at room temperature for 2 h. Following three washes with ice-cold lysis buffer, the beads were pelleted and resuspended in SDS‒PAGE loading buffer. Finally, the immunoprecipitated samples were denatured by boiling at 100 °C for 5 min prior to further analysis.

### GST pull-down assays

Recombinant GST-AR and His-STARD4 proteins were expressed in BL21 (Rosetta) bacterial cells and purified using the standard protocol provided with a protein purification kit (Biolinkedin, Shanghai, China). A GST pull-down assay was performed using a GST protein interaction pull-down kit (Biolinkedin, Shanghai, China) in accordance with the manufacturer's instructions. Briefly, purified GST-tagged proteins were immobilized onto glutathione-Sepharose beads, which were then incubated with His-tagged proteins in PMSF-supplemented affinity isolation buffer. After incubation, the protein-bound beads were magnetically separated, washed, and the captured complexes were analyzed by western blotting.

### Immunofluorescence staining

Experimentally treated cells were seeded onto glass coverslips in 12-well culture plates at a density of 1.5 × 10^5^ cells per well. The cells were rinsed with PBS and subsequently fixed with 4% paraformaldehyde for 30 min at room temperature. After fixation, the cells were permeabilized with 0.5% Triton X-100 for 15 min. The cells were then rinsed with PBS for 15 min and blocked with goat serum (ZSGB-Bio, Beijing, China) at room temperature. Cells were then incubated with primary antibodies overnight at 4 °C, followed by PBS rinsing and incubation with fluorophore-conjugated secondary antibodies. Finally, samples were washed twice with PBS, counterstained with DAPI for nuclear visualization, and imaged using a Zeiss LSM880 confocal microscope. Antibody details are provided in Supplementary Table 2.

### ER tracker staining assay

Stably transfected cells were seeded in Petri dishes for confocal microscopy and rinsed twice with a balanced salt solution enriched with Ca^2^⁺ and Mg^2^⁺. Next, the cells were incubated with prewarmed ER-Tracker Red Kit working solution (Beyotime) for 15 min, followed by two washes with RPMI 1640 medium to eliminate surplus dye. Then, the cells were fixed with a 4% paraformaldehyde solution for 5 min and counterstained with DAPI to visualize the nuclear architecture. Images were acquired through a confocal microscope.

### ORO staining

ORO staining was conducted using both cells and tissue sections and an ORO stain kit (Servicebio, Wuhan, China), adhering meticulously to the manufacturer’s guidelines. The ORO working solution was prepared by combining six parts of ORO stock solution with four parts of deionized water and subsequently purifying the mixture through a 0.22 µm syringe filter. Cells in 6-well plates were fixed with 4% paraformaldehyde at ambient temperature for 10 min and subsequently rinsed with 60% isopropanol before being stained with ORO solution in a light-protected environment for 30 min at room temperature. Prior to microscopic examination, the cells were differentiated in 60% isopropanol for 5 s and subsequently washed with deionized water. Tissues were embedded in optimal cutting temperature compound, sectioned and fixed in 4% paraformaldehyde for 10 min. The sections were incubated in ORO staining solution under light-protected conditions for 10 min at room temperature, followed by counterstaining with haematoxylin to delineate the nuclear structures. Observations and photographic documentation were carried out using a microscope. ImageJ software was used to measure the diameter of lipid droplets within cells. Subsequently, ORO was redissolved in isopropanol, and the absorbance was measured at 570 nm.

### Triglyceride (TG), cholesterol, and testosterone measurements

Ntracellular triglyceride (TG) and total cholesterol levels were quantified using commercial assay kits (Nanjing Jiancheng Bioengineering Institute, Nanjing, China), respectively. Cells were lysed in normal saline via ultrasonic disruption. For the assays, 250 μL of each lysate was mixed with 2.5 μL of the kit-specific working solution in a 96-well plate, followed by incubation at 37 °C for 10 min in the dark to avoid photodegradation. Optical density was measured at 510 nm using an ELISA reader. Additionally, testosterone concentrations were determined using an ELISA kit (Beyotime Biotechnology) following the manufacturer’s protocol.

### Bioinformatics analysis

Gene expression data and corresponding clinical information pertaining to a PCa cohort were procured from The Cancer Genome Atlas (TCGA) database and the Gene Expression Omnibus (GEO) database. The differential expression of STARD4 between malignant tissues and adjacent nontumorous tissues was assessed utilizing the Wilcoxon test. Kaplan‒Meier survival analysis was used to elucidate the correlation between STARD4 expression levels and patient survival outcomes. Furthermore, datasets from DKFZ and MSKCC were utilized to investigate the associations between STARD4 expression and both pathological T stage and Gleason score in PCa. To reveal the biological functions of STARD4, gene set enrichment analysis (GSEA) was conducted to identify the pathways that were significantly affected. Additionally, the UbiBrowser database was used to predict potential E3 ubiquitin ligases that interact with the AR protein.

### LC–MS analysis

For untargeted lipidomics analysis, control and STARD4-overexpressing 22Rv1 cells were snap-frozen in liquid nitrogen immediately after collection. Lipids were extracted using a dichloromethane/methanol mixture (1:1, v/v), followed by centrifugation (16,000 × g, 15 min, 4 °C) to pellet insoluble debris. The supernatants were subjected to chromatographic separation using a Vanquish HPLC system (Thermo Fisher Scientific) with a Phenomenex Kinetex C18 column (2.1 mm × 100 mm, 2.6 μm). Mass spectrometry data were acquired using an Orbitrap Exploris 120 mass spectrometer (Thermo Fisher Scientific). Data analysis was performed using the CentWave algorithm in XCMS, and lipid identification was conducted by searching against the LipidBlast 2022 database.

### Animal studies

All animal experiments were approved by the First Affiliated Hospital of Shihezi University Animal Care Committee (Document No. A2024-038–01). Five-week-old male athymic nude mice were procured from SIPEIFU Biotechnology (Beijing, China). To explore the influence of STARD4 on PCa growth, a suspension of 3 × 10^6^ 22Rv1 cells with STARD4 overexpression or depletion was subcutaneously injected into mice (n = 5). Tumour progression was monitored every four days until the mice were sacrificed after 32 days, after which the tumours were excised for subsequent immunohistochemical staining. Moreover, to assess the effect of STARD4 on enzalutamide resistance in PCa, 2 × 10^6^ C4-2-EnzR cells transfected with either an empty vector or a STARD4-oe vector were implanted subcutaneously into mice. Four days postinoculation, the mice were segregated into four groups (*n* = 5): vector, vector + enzalutamide (10 mg/kg, p.o.), STARD4-oe, and STARD4-oe + enzalutamide (10 mg/kg, p.o.). Following a treatment period of 28 days, the mice were humanely euthanized, and the tumours were collected for subsequent analysis.

### Statistical analysis

All the statistical analyses were conducted using R version 4.3.0 and GraphPad Prism version 9. For comparisons between two groups, normally distributed data were analysed for statistical significance using Student’s t test, whereas nonnormally distributed data were assessed with the Mann–Whitney U test. One-way ANOVA and two-way ANOVA followed by Tukey’s multiple comparison test was used to compare multiple groups. Spearman correlation was used to conduct all correlation analyses. Univariate Cox regression analysis was employed to examine the relationships between the expression levels of candidate genes and patient survival outcomes. Survival analysis involved the comparison of Kaplan‒Meier curves utilizing the log-rank test. All assays were conducted at least three times,and data are presented as means ± standard deviations. Statistical significance was defined as a p value less than 0.05.

## Results

### The downregulation of STARD4 expression is closely linked to PCa progression and poor prognosis of PCa patients

**Fig. 1:**
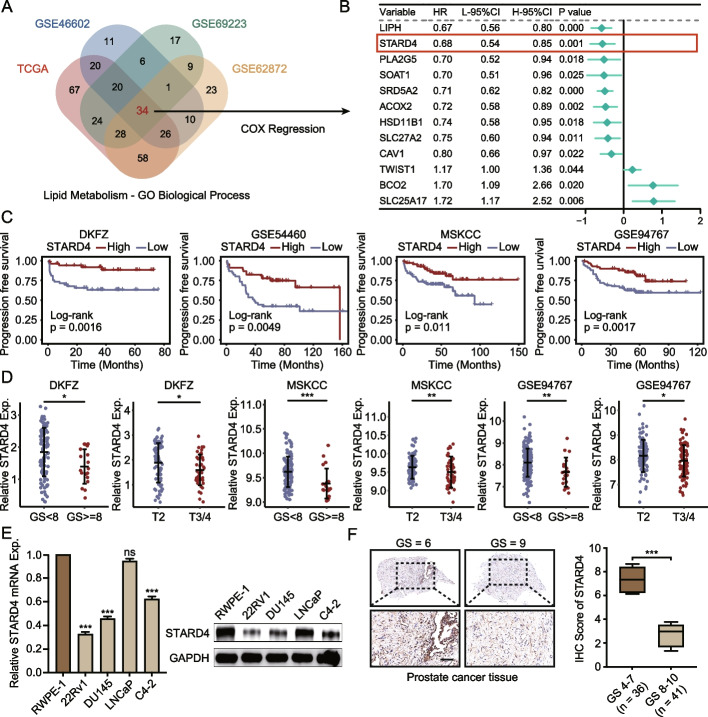
Decreased STARD4 expression in PCa. **A** Screening of lipid metabolism‐related genes in the TCGA PCa database and three different PCa transcriptome datasets. **B** Univariate Cox regression analysis was performed using PCa progression risk- and lipid metabolism-related genes to screen for the critical lipid metabolism gene STARD4. **C** Analysis of Kaplan‒Meier progression-free survival curves for STARD4^high^ and STARD4^low^ patients in the DKFZ, GSE54460, MSKCC, and GSE94767. The log-rank test was used for comparison. **D** Correlation of STARD4 expression with the Gleason score and tumour stage according to an in-depth analysis of the DKFZ, MSKCC, and GSE94767 datasets. **E** Lysates of RWPE-1, 22Rv1, DU145, LNCaP, and C4-2 cells were harvested, and the mRNA and protein levels of STARD4 were assessed via RT‒qPCR and western blotting(*n* = 3). **F** STARD4 expression evaluation by IHC assays in PCa tissues from 77 patients. Scale bar: 100 μm. Unpaired two-tailed Student’s t test (**D**, **F**); One-way ANOVA (**E**). (ns, not significant; *, *p* < 0.05; **, *p* < 0.01; ***, *p* < 0.001). Data are presented as the mean ± SD

To identify lipid metabolism genes that play significant roles in PCa, we analysed differentially expressed genes related to lipid metabolism between PCa and normal tissues using four transcriptomic datasets (TCGA-PRAD, GSE62872, GSE46602, and GSE69223). This process led to the identification of 34 genes of interest (Fig. 1A, S1A). Further screening with univariate Cox regression identified 12 genes that were strongly associated with PCa prognosis (Fig. 1B). Notably, STARD4 expression was found to be significantly downregulated in PCa, and high STARD4 expression was linked to a reduced risk of PCa (Fig. 1B, S1B). Kaplan–Meier survival analysis revealed that patients with low STARD4 expression had significantly shorter progression-free survival than those with high STARD4 expression did (Fig. 1C). Additionally, low STARD4 expression was correlated with tumour progression, metastasis, and high Gleason scores (Fig. 1D).

To validate these findings, we assessed STARD4 expression levels in several PCa cell lines. Our observations indicated that STARD4 levels were significantly lower in PCa cells than in the human prostatic epithelial cell line RWPE-1 (Fig. 1E). This observation was corroborated by analysis of publicly available data from the HPA database, which revealed significantly higher STARD4 expression in benign prostate tissues relative to PCa (Figure S1C). Furthermore, IHC staining of the PCa specimens demonstrated that STARD4 protein levels were substantially lower in tumors with high Gleason scores (*n* = 41) than in those with low Gleason scores (*n* = 36) (Fig. 1F). Collectively, these results suggest that low STARD4 expression is associated with PCa progression and poor patient survival outcomes.

### STARD4 suppresses cell growth and xenograft progression

**Fig. 2:**
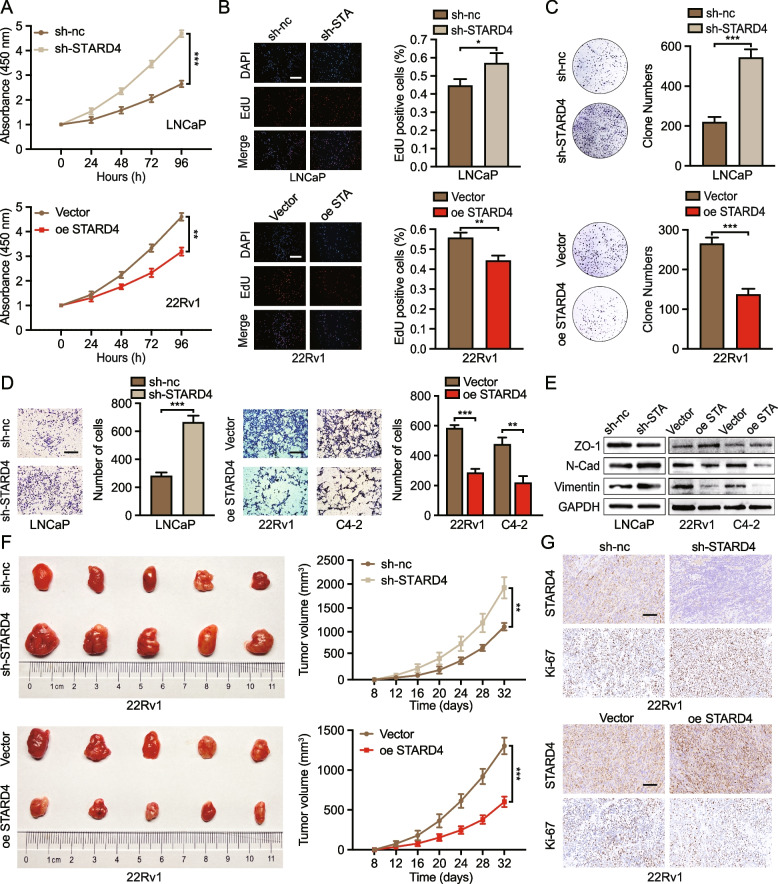
STARD4 overexpression suppresses PCa cell growth and xenograft progression. **A** Cell viability was assessed by CCK-8 assay in LNCaP cells with STARD4 knockdown and 22Rv1 cells with STARD4 overexpression (*n* = 3). **B-C** Cell proliferation was determined by EdU and colony formation assays using LNCaP cells infected with STARD4-knockdown or 22Rv1 cells infected with STARD4-overexpressing plasmids (*n* = 3). Scale bar: 50 μm. **D** Evaluation of cell migration in LNCaP, 22Rv1, and C4-2 cells via Transwell assays (*n* = 3). Scale bar: 50 μm. **E** Expression of EMT markers in STARD4-knockdown LNCaP cells or STARD4-overexpressing 22Rv1 and C4-2 cells. **F** Xenograft PCa tumour growth upon subcutaneous implantation of transfected 22Rv1 cells into nude mice (*n* = 5). Tumour size was monitored every four days, and the tumours were photographed at the endpoint. The growth curves for the cumulative differences in growth under the different treatments are shown. **G** IHC staining of STARD4 and Ki-67 in tumours from different groups. Scale bar: 100 μm. Unpaired two-tailed Student’s t test (**B, C, D**); Two-way ANOVA (**A, F**). (*, *p* < 0.05; **, *p* < 0.01; ***, *p* < 0.001). Data are presented as the mean ± SD

To elucidate the biological function of STARD4 in PCa, we overexpressed STARD4 in the 22Rv1 and C4-2 cell lines while employing shRNA to achieve stable STARD4 depletion in LNCaP, 22Rv1, and C4-2 cells (Figure S2A). The impact of STARD4 on cell proliferation was investigated through CCK-8 and EdU assays. Our results demonstrated that STARD4 overexpression markedly suppressed cell proliferation, whereas STARD4 silencing enhanced this process (Fig. 2A-B, S2B-C). Furthermore, colony formation assays confirmed that STARD4 overexpression significantly diminished the proliferative ability of PCa cells (Fig. 2C, S2D).

Moreover, transwell assays revealed that the increased expression of STARD4 inhibited the migration of 22Rv1 and C4-2 cells, whereas the knockdown of STARD4 in LNCaP cells promoted their migratory ability (Fig. 2D). Subsequent immunoblot analysis of epithelial‒mesenchymal transition (EMT)-related markers revealed that, compared with control cells, cells overexpressing STARD4 presented reduced levels of N-cadherin and vimentin and an increased level of ZO-1 (Fig. 2E). To validate these findings in vivo, xenograft models were established. STARD4 overexpression in PCa models significantly diminished the tumour growth rate, size, and weight (Fig. 2F, S2E). Consistent with these observations, IHC staining for Ki-67 confirmed the impact of STARD4 on proliferation in PCa xenografts (Fig. 2G, S2F). In summary, both the in vitro and in vivo results strongly demonstrated that STARD4 markedly suppresses PCa cell proliferation and restrains xenograft tumour progression.

### STARD4 inhibits abnormal lipid accumulation in PCa

**Fig. 3:**
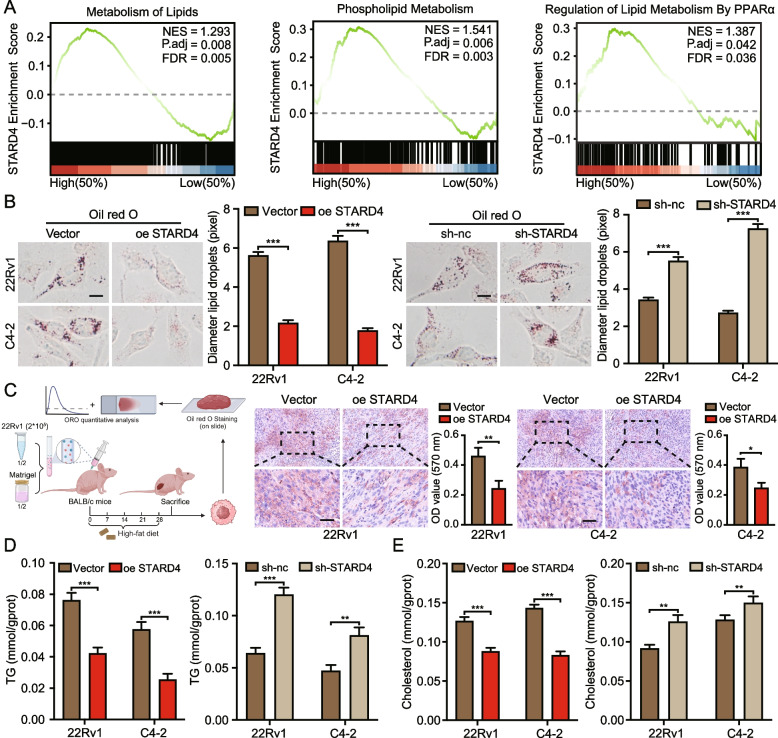
STARD4 modulates abnormal lipid accumulation in vivo and in vitro. **A** GSEA was carried out to identify the related pathways that were significantly enriched in the STARD4^high^ group. **B** ORO staining, a direct indicator of lipid accumulation, was performed in 22Rv1 and C4-2 cells (*n* = 3). Scale bar: 10 μm. **C** 22Rv1 or C4-2 cells were mixed with Matrigel and injected into the dorsal region of nude mice. Graphical representation and representative images of ORO staining of xenograft tumour sections from the vector and STARD4 overexpression groups (*n* = 5). Scale bar: 50 μm. **D-E** TG and cholesterol contents were assessed as quantitative indicators of lipid accumulation in 22Rv1 or C4-2 cells (*n* = 3). Unpaired two-tailed Student’s t test (**B, C, D, E**). (*, *p* < 0.05; **, *p* < 0.01; ***, *p* < 0.001). Data are presented as the mean ± SD

To investigate the role of STARD4 in lipid metabolism in PCa, the TCGA-PRAD cohort was utilized to perform GSEA. The findings revealed a significant association between STARD4 expression and lipid metabolic processes in PCa (Fig. 3A, S3A). To validate this observation, ORO staining was employed to assess lipid accumulation within PCa cells. As depicted in Fig. 3B, compared with control cells, PCa cells overexpressing STARD4 presented a marked reduction in lipid droplet (LD) density, whereas a significant increase in LD density was noted in STARD4-knockdown cells. These results support the hypothesis that STARD4 plays a crucial role in modulating lipid metabolism in PCa.

To elucidate the impact of STARD4 on lipid accumulation in vivo, Matrigel mixed with PCa 22Rv1 or C4-2 cells was implanted subcutaneously into mice. Consistent with the in vitro findings, xenograft tumours with upregulated STARD4 expression presented reduced lipid accumulation within the cytoplasm, whereas STARD4 silencing led to a significant increase in lipid accumulation (Fig. 3C, S3B). To comprehensively characterize these lipid alterations, we performed untargeted LC–MS lipidomic profiling of STARD4-overexpressing versus control 22Rv1 cells. This analysis identified substantial decreases in multiple lipid classes, including cholesteryl esters (CE), triglycerides (TG), hexosylceramides (HexCer), and phosphatidylcholines (PC) (Figure S3C). Pathway analysis of these differentially regulated lipid species revealed significant enrichment of key metabolic processes, most notably fatty acid transport, triglyceride homeostasis, and cholesterol metabolism (Figure S3D), providing mechanistic insight into STARD4's role in lipid regulation. Based on this, TG and cholesterol contents were measured to quantify lipid accumulation in PCa cells. Consistent with the lipidomics data, stable overexpression of STARD4 resulted in lower TG and cholesterol levels (Fig. 3D-E). Collectively, these findings suggest that the upregulation of STARD4 expression mitigates lipid accumulation in PCa.

### STARD4 promotes cell apoptosis by inducing ER stress

**Fig. 4:**
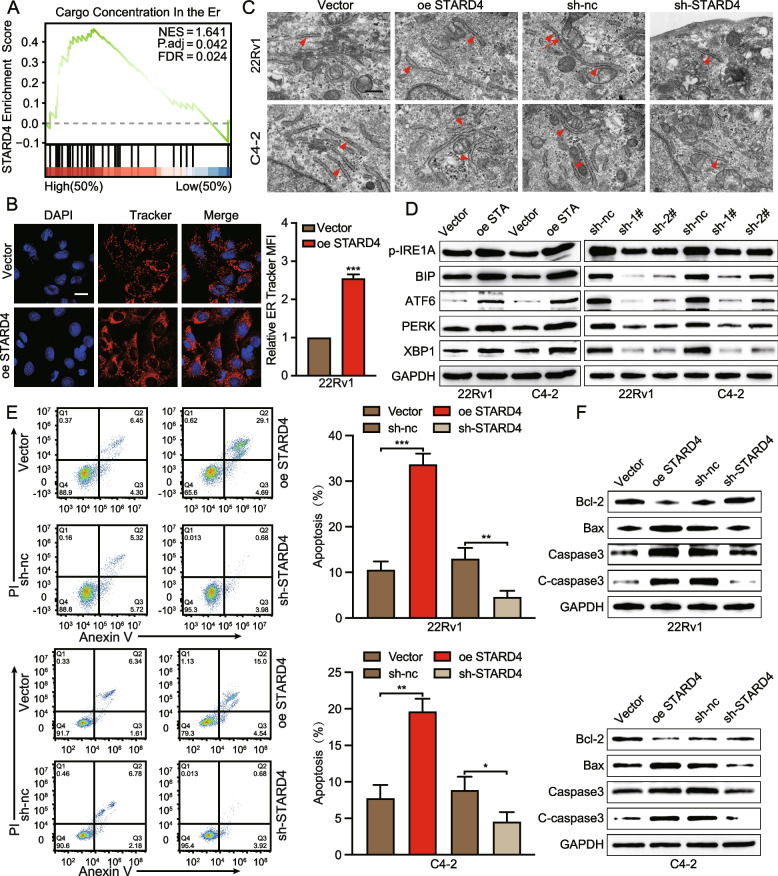
STARD4 promotes ER stress-mediated cell apoptosis. **A** GSEA revealed that the cargo concentration in the ER was significantly increased in PCa cells with high STARD4 expression. **B** ER structural changes in 22Rv1 cells transfected with a vector or STARD4 expression plasmid were observed via ER Tracker (*n* = 3). Scale bar: 10 μm. **C** Analysis of the ER structure in STARD4-overexpressing or STARD4-knockdown 22Rv1 and C4-2 cells by TEM. Scale bar: 1 μm. **D** Protein levels of ER stress markers (p-IRE1A, BIP, ATF6, PERK, and XBP1) in 22Rv1 and C4-2 cells with stable overexpression or knockdown of STARD4. **E** The percentage of apoptotic 22Rv1 and C4-2 cells with STARD4 overexpression or knockdown was determined by flow cytometry (*n* = 3). **F** Western blot analysis of apoptosis-related markers (Bcl-2, Bax, Caspase3, and C-caspase3) in 22Rv1 and C4-2 cells with STARD4 overexpression or knockdown. Unpaired two-tailed Student’s t test (**B, E**). (*, *p* < 0.05; **, *p* < 0.01; ***, *p* < 0.001). Data are presented as the mean ± SD

Previous studies have shown that LDs alleviate the ER stress response, thereby promoting ER homeostasis and increasing the viability of tumour cells. Furthermore, our GSEA results revealed a significant association between high STARD4 expression and the signalling pathways associated with the cargo concentration within the ER (Fig. 4A). Given that STARD4 inhibits abnormal lipid accumulation in PCa, we investigated whether it also impacts ER stress in PCa cells. ER tracking imaging analysis revealed ER dilation in cells overexpressing STARD4 (Fig. 4B), a finding corroborated by TEM, which demonstrated the presence of abnormally dilated and irregularly shaped rough ER in cells with upregulated STARD4 expression (Fig. 4C). Additionally, the overexpression of STARD4 in 22Rv1 and C4-2 cells was associated with increased levels of ER stress markers; in contrast, STARD4 knockdown in these cells led to the decreased expression of these markers (Fig. 4D, S4A-B). These observations indicate that STARD4 plays a role in modulating ER stress in PCa cells. Given that ER stress frequently triggers cell death through the activation of apoptotic signalling pathways [27, 28], flow cytometry was used to assess the degree of apoptosis. Consistent with the functional predictions, STARD4 overexpression in the 22Rv1 and C4-2 cell lines significantly increased the apoptotic indices, whereas STARD4 silencing conferred resistance to apoptosis (Fig. 4E). Immunoblotting revealed that the STARD4-mediated downregulation of Bcl-2 (antiapoptotic) expression was concurrent with upregulated Bax expression and increased caspase-3 cleavage (Fig. 4F), mechanistically validating the ability of STARD4 to induce apoptosis in PCa.

### STARD4 regulates the AR signalling pathway by affecting androgen levels and AR protein stability

**Fig. 5:**
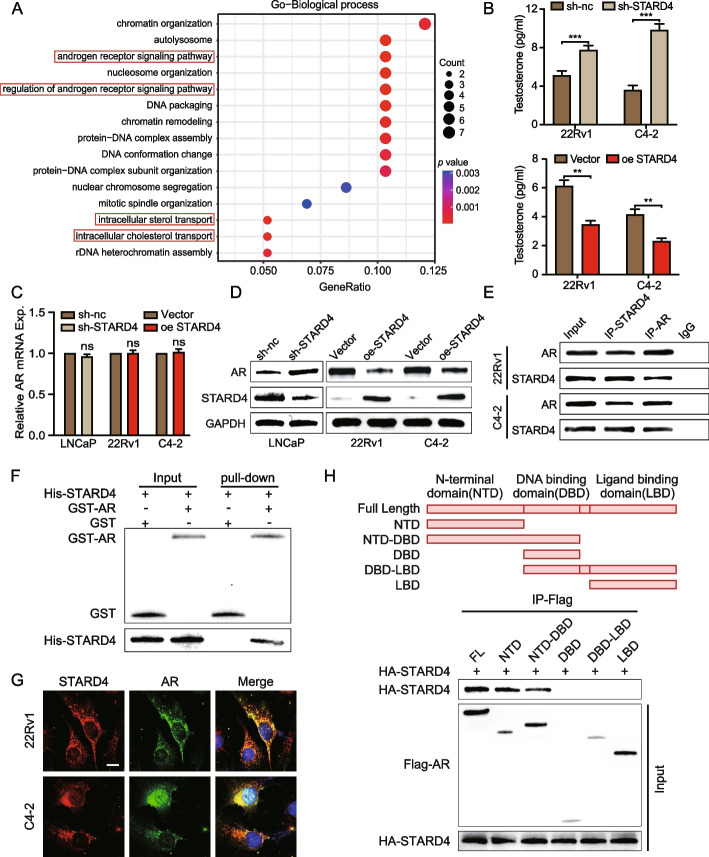
STARD4 affects the AR signalling pathway by affecting androgen levels and AR protein stability. **A** Transcriptome sequencing of 22Rv1 cells stably overexpressing STARD4 (*n* = 3). GO enrichment analysis of the differentially expressed genes. **B** Measurement of testosterone content to assess androgen levels in 22Rv1 and C4-2 cells with stable overexpression or knockdown of STARD4 (*n* = 3). **C‒D** Assessment of AR mRNA and protein levels in LNCaP cells with stable STARD4 knockdown and 22Rv1 and C4‒2 cells with stable STARD4 overexpression via RT‒qPCR and western blotting (*n* = 3). **E** Co-IP analysis using 22Rv1 and C4-2 cell whole-cell lysates with STARD4, AR or IgG antibodies, followed by western blotting with the appropriate antibodies. **F** GST affinity-isolation assay with purified tagged STARD4 and AR proteins in 293 T cells, followed by western blot analysis. **G** Co-transfection of 22Rv1 and C4-2 cells with STARD4 and AR overexpression plasmids and subsequent immunofluorescence staining with anti-STARD4 and anti-AR antibodies. Scale bar: 10 μm. **H** Co-IP assays were conducted using 293T cells transfected with HA-labelled STARD4, Flag-labelled AR, or structural domain plasmids (Flag-AR-NTD, Flag-AR-NTD-DBD, Flag-AR-DBD, Flag-AR-DBD-LBD, or Flag-AR-LBD). Unpaired two-tailed Student’s t test (**B, C**). (ns, not significant; *, *p* < 0.05; **, *p* < 0.01). Data are presented as the mean ± SD

To elucidate the regulatory mechanisms of STARD4 in PCa, we performed RNA-seq analysis using 22Rv1 cells stably overexpressing STARD4. Gene ontology analysis of the differentially expressed genes revealed that STARD4 overexpression significantly affected the AR signalling pathway, regulation of the AR signalling pathway, intracellular sterol transport, and intracellular cholesterol transport (Fig. 5A). Considering that increased intratumoural androgen synthesis is a critical factor in PCa progression and that cholesterol serves as a vital precursor for androgen synthesis, we hypothesized that STARD4 influences androgen levels within tumour cells. Consequently, we assessed androgen synthesis in these cells. The results indicated that the upregulation of STARD4 expression significantly reduced testosterone levels, whereas the downregulation of STARD4 expression led to a significant increase in testosterone content. These findings suggest that STARD4 inhibits intratumoural androgen synthesis (Fig. 5B), thereby playing a crucial role in modulating hormone-dependent pathways in PCa.

Notably, androgens exert their physiological effects by binding to and activating the AR, with AR expression being a critical oncogenic driver at various stages of PCa development and progression [29]. To better understand the relationship between STARD4 and AR, we examined both the mRNA and protein levels of AR in PCa cells overexpressing STARD4 or with STARD4 depletion. RT‒qPCR analysis revealed no significant changes in AR mRNA levels across these conditions (Fig. 5C). However, western blot analysis revealed that STARD4 overexpression led to decreased AR protein levels, whereas STARD4 knockdown resulted in increased AR protein levels compared with those in the controls (Fig. 5D, S5A). This discrepancy between the AR mRNA and protein levels suggested that STARD4 might regulate AR levels through mechanisms affecting protein stability rather than transcription. To test this hypothesis, we investigated whether STARD4 interacts with AR. Co-IP experiments confirmed an interaction between STARD4 and AR (Fig. 5E). Additional evidence for a direct interaction was provided by GST pull-down assays, which demonstrated that AR directly binds to the STARD4 protein (Fig. 5F). IHC staining supported these findings by revealing the colocalization of STARD4 and AR within the cytoplasm (Fig. 5G). The AR protein comprises three structural modules: an N-terminal transactivation domain (NTD), a central DNA-binding domain (DBD), and a C-terminal ligand-binding domain (LBD) [30]. To map STARD4-AR interaction interfaces, HEK293T cells were transfected with AR domain-specific deletion constructs. Co-IP assays revealed that STARD4 binds to the AR-NTD and full-length AR but not to the DBD or LBD truncations (Fig. 5H), establishing the NTD as the critical interaction interface.

**Fig. 6:**
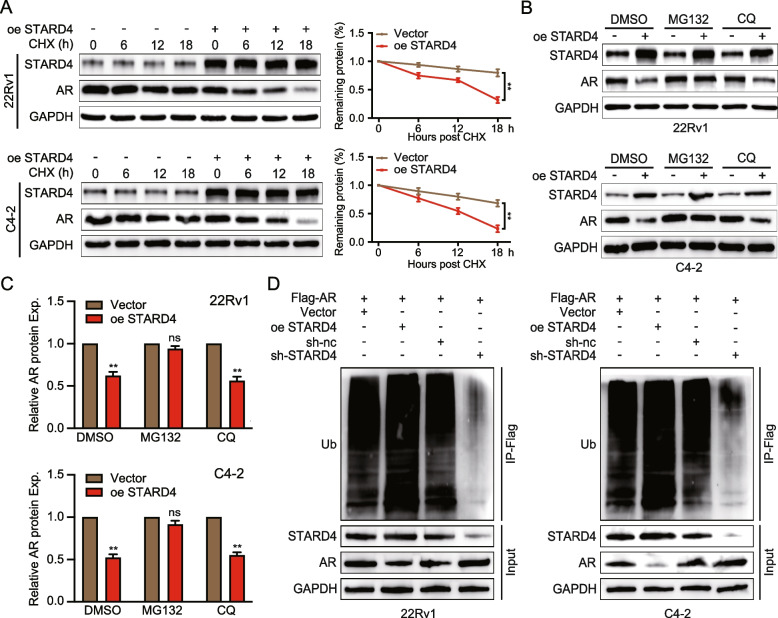
STARD4 affects the stability of the AR protein via the ubiquitin‒proteasome degradation pathway. **A** Protein synthesis inhibition in 22Rv1 and C4-2 cells with STARD4 overexpression using CHX (10 μM), with AR protein levels determined by western blotting at 0, 6, 12, and 18 h (*n* = 3). **B-C** Treatment of 22Rv1 and C4-2 cells stably overexpressing STARD4 with DMSO, chloroquine (CQ, 20 μM), or MG132 (50 μM) and analysis of AR protein levels by western blotting (*n* = 3). **D** Assessment of AR ubiquitination levels in 22Rv1 and C4-2 cells stably overexpressing STARD4. Immunoprecipitation of the cells was performed with anti-Flag-AR antibodies, followed by western blotting with the indicated antibodies. Unpaired two-tailed Student’s t test (**C**); Two-way ANOVA (**A**). (ns, not significant; **, *p* < 0.01). Data are presented as the mean ± SD

To validate the hypothesis that STARD4 regulates AR protein stability, cell lines in different groups were treated with cycloheximide (CHX), an inhibitor of protein synthesis, to assess AR protein turnover over time. The results revealed that the half-life of AR was significantly shortened after STARD4 overexpression (Fig. 6A). Conversely, STARD4 knockdown prolonged the half-life of AR (Figure S5B). To explore the mechanism by which STARD4 regulates AR protein expression, PCa cells with the stable overexpression or knockdown of STARD4 were treated with a proteasome inhibitor (MG132) and a lysosome inhibitor (chloroquine). Western blot analysis revealed that only MG132 reversed the reduction in AR protein levels caused by STARD4 overexpression in PCa cells (Fig. 6B-C, Figure S5C). These findings indicate that STARD4 affects AR protein stability through the ubiquitin‒proteasomal degradation pathway rather than via lysosomal degradation. To test this hypothesis, immunoprecipitation was subsequently performed to measure the ubiquitination level of AR. The results revealed a significant increase in AR ubiquitination in PCa cells overexpressing STARD4 (Fig. 6D). These findings suggest that STARD4 enhances the ubiquitination and subsequent proteasomal degradation of AR, thereby regulating its protein stability.

### STARD4 regulates the protein stability of AR by affecting the interaction between UBE4B and AR

**Fig. 7:**
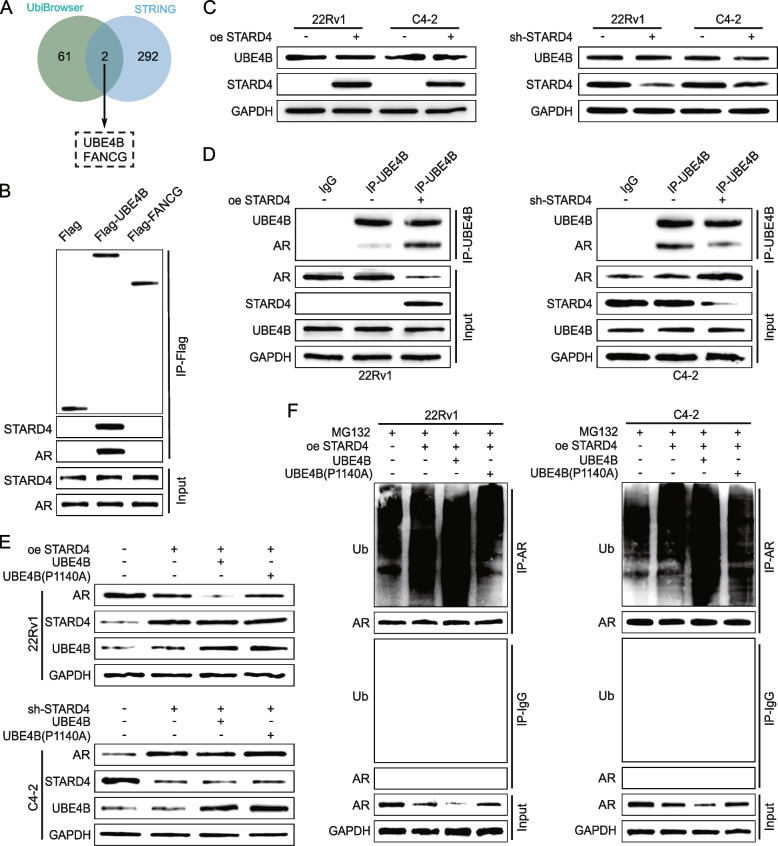
STARD4 promotes AR protein ubiquitination and degradation by enhancing the interaction between UBE4B and AR**. A** Venn diagram illustrating potential ubiquitinating enzymes that regulate the ubiquitin-mediated degradation of AR. **B** 22Rv1 cell lysates were immunoprecipitated with an anti-Flag antibody and then subjected to western blotting with the indicated antibodies to examine the interaction between the two ubiquitinating enzymes and STARD4 or AR. **C** The protein levels of UBE4B in PCa cells were determined in 22Rv1 and C4-2 cells with STARD4 overexpression or knockdown. **D** 22Rv1 cells were transfected with a STARD4 overexpression plasmid, and C4-2 cells were transfected with a STARD4 knockdown plasmid. Co-IP with IgG or an anti-UBE4B antibody was followed by western blotting with the indicated antibodies. **E** In 22Rv1 cells, a STARD4 overexpression plasmid was transfected alone or in combination with a UBE4B wild-type or mutant UBE4B (P1140A) plasmid, whereas in C4-2 cells, a STARD4 knockdown plasmid was transfected alone or in combination with the above two UBE4B plasmids. Western blotting was performed with the indicated antibodies. **F** 22Rv1 and C4-2 cells were transfected with a STARD4 overexpression plasmid and treated with MG132. Protein lysates were collected from the cells to perform Co-IP with IgG or an anti-AR antibody, followed by western blotting with the indicated antibodies

To elucidate the mechanism by which STARD4 regulates AR protein ubiquitination, computational tools such as STRING and Ubibrowser were used to predict potential ubiquitinases that could bind to AR. This screening process identified two candidate ubiquitinase genes: UBE4B and FANCG (Fig. 7A). Subsequent Co-IP experiments confirmed that only UBE4B interacts with both STARD4 and AR (Fig. 7B), suggesting a specific role for UBE4B in this context. UBE4B is an E3 ubiquitin ligase known for its involvement in the ubiquitination and degradation of protein substrates [31], playing a significant role in tumorigenesis and progression across various types of cancer [32–35]. Given this background, subsequent experiments were conducted to determine whether UBE4B acts as the key E3 ubiquitin ligase that mediates STARD4-induced AR degradation. Surprisingly, altering STARD4 expression did not affect the mRNA or protein levels of UBE4B (Fig. 7C, S6A). However, Co-IP assays revealed that the interaction between AR and UBE4B was enhanced by STARD4 overexpression in PCa cells but was reduced when STARD4 was depleted (Fig. 7D, S6B). These findings indicate that STARD4 does not affect the expression level of UBE4B but instead enhances the interaction between UBE4B and AR, facilitating the ubiquitination and subsequent degradation of AR. These findings highlight the critical role of STARD4 in modulating AR stability through its interactions with UBE4B rather than by directly regulating UBE4B expression levels. Given that UBE4B ubiquitination activities are dependent on a highly conserved proline at position 1140 and that the mutant form of UBE4B (1140A) cannot increase the ubiquitination of target proteins [36], experiments were conducted to assess the importance of UBE4B ubiquitination activity for STARD4-mediated AR protein stability. The results showed that wild-type UBE4B, not the UBE4B (1140A) mutant, restored AR protein degradation induced by STARD4 overexpression and reversed the upregulated AR expression caused by STARD4 knockdown (Fig. 7E). Consistently, the upregulation of STARD4 expression increased AR ubiquitination, and this effect was compromised when UBE4B was inhibited or mutated, indicating that the ubiquitination activity of UBE4B is essential for the STARD4-mediated regulation of AR stability (Fig. 7F). These findings underscore the indispensable role of UBE4B in mediating the effects of STARD4 on AR, particularly highlighting the necessity of the enzymatic activity of UBE4B for this regulatory process.

### STARD4 regulates the sensitivity of PCa to enzalutamide

**Fig. 8:**
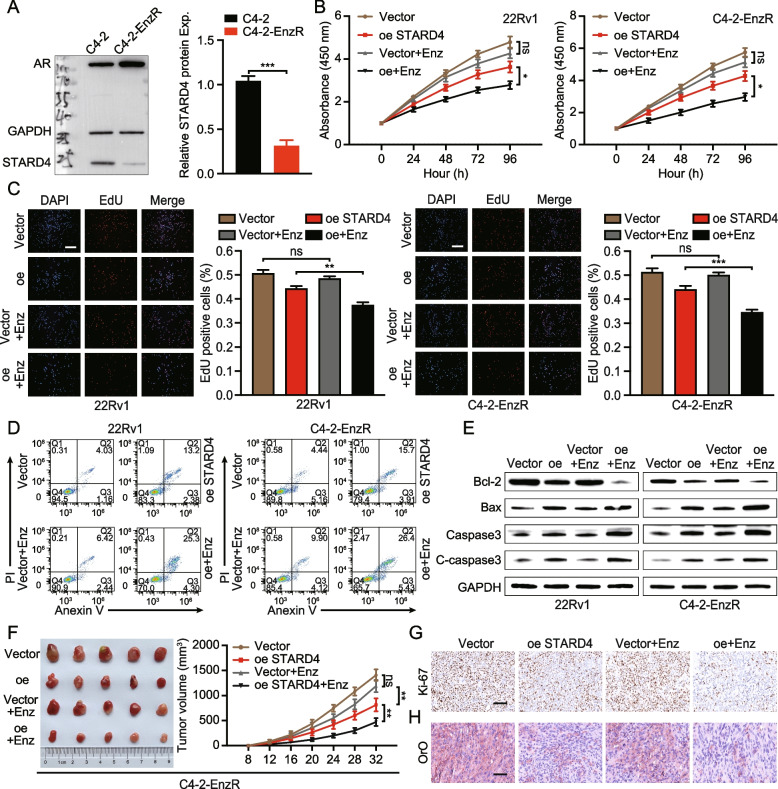
STARD4 modulates enzalutamide sensitivity in PCa. **A** Western blotting was performed to examine the protein expression of STARD4 and AR in C4-2 and C4-2-EnzR cells (*n* = 3). **B** 22Rv1 and C4-2-EnzR cells stably overexpressing STARD4 were treated with DMSO or enzalutamide, and cell viability was assessed using the CCK-8 assay (*n* = 3). **C** Evaluation of cell proliferation in vector and STARD4-overexpressing 22Rv1 or C4-2-EnzR cells treated with DMSO or enzalutamide via an EdU incorporation assay (*n* = 3). Scale bar: 50 μm. **D** Cell apoptosis in vector and STARD4-overexpressing 22Rv1 or C4-2-EnzR cells treated with DMSO or enzalutamide was assessed by flow cytometry. **E** The expression of apoptosis-related markers (Bcl-2, Bax, Caspase3, and C-caspase3) in 22Rv1 or C4-2-EnzR cells was assessed by western blotting. **F** Nude mice bearing C4-2-EnzR xenografts with stable STARD4 overexpression were treated with vehicle control or enzalutamide (10 mg/kg, p.o.) for 28 days (*n* = 5). Tumour size was monitored every four days, and the tumours were weighed and photographed at the endpoint. **G** IHC staining for Ki-67 in tumours from the four indicated groups for apoptosis analysis. Scale bar: 100 μm. **H** ORO staining of tumours to analyse lipid accumulation in the four indicated groups. Scale bar: 50 μm. Unpaired two-tailed Student’s t test (**A, C**); Two-way ANOVA (**B, F**). (ns, not significant; *, *p* < 0.05; **, *p* < 0.01). Data are presented as the mean ± SD

Given the critical role of the AR signalling pathway in the effectiveness of antiandrogen therapy, this investigation was extended to explore the role of STARD4 in modulating enzalutamide resistance in PCa. An enzalutamide-resistant C4-2 cell model was established. Compared with their enzalutamide-sensitive counterparts, C4-2-EnzR cells presented reduced sensitivity to enzalutamide (Figure S7A). Western blot analysis revealed that STARD4 expression was decreased while AR expression was increased in C4-2-EnzR cells (Fig. 8A). Additionally, ORO staining revealed an increase in LD size in C4-2-EnzR cells relative to that in C4-2 cells (Figure S7B). Subsequent experiments were conducted to assess the impact of STARD4 on cell proliferation and the response to enzalutamide treatment. CCK-8 and EdU assay results demonstrated that PCa cells with STARD4 depletion were resistant to enzalutamide treatment, whereas increased STARD4 expression enhanced the suppressive effects of enzalutamide on the proliferation of both 22Rv1 and C4-2-EnzR cells (Fig. 8B-C). Flow cytometry analysis was used to evaluate cell apoptosis following enzalutamide treatment. STARD4 overexpression restored the sensitivity of 22Rv1 and C4-2-EnzR cells to enzalutamide-induced apoptosis (Figs. 8D and S7C). Western blotting confirmed these findings, showing that STARD4 overexpression promoted apoptosis in PCa cells treated with enzalutamide (Fig. 8E).

To assess the therapeutic efficacy of STARD4 in a setting that closely mimics the biological environment of PCa more accurately, an in vivo study was conducted using a mouse model. C4-2-EnzR cells, either with a control vector or stably overexpressing STARD4, were transplanted subcutaneously into mice. The results showed that enzalutamide treatment alone had minimal effects on tumour growth. In contrast, STARD4 overexpression led to the significant inhibition of tumour growth when it was combined with enzalutamide treatment (Fig. 8F-G, S7D-E). Additionally, ORO staining revealed that, compared with the other treatments, the combination of STARD4 overexpression and enzalutamide treatment resulted in a significant reduction in LD size within tumour tissues (Fig. 8H, S7F). Collectively, these findings indicate that STARD4 has the potential to serve as a therapeutic target for treating PCa, particularly in overcoming acquired resistance to enzalutamide.

## Discussion

Endocrine therapy, which targets the AR signalling pathway, is fundamental for treating advanced PCa [37]. This therapeutic approach has evolved from gonadal testosterone deprivation to include methods that block the synthesis of adrenal and other extragonadal androgens, as well as direct AR inhibition. Despite these advancements, resistance to endocrine therapy inevitably develops, necessitating the exploration of new therapeutic strategies [38, 39]. Recent research highlights the significant role of lipid and cholesterol metabolism in PCa progression and the development of CRPC [40]. Cholesterol not only increases intratumoural androgen production but is also elevated in enzalutamide-resistant CRPC cells [41]. Given this context, there is a critical need to identify candidate genes related to lipid metabolism that could influence PCa progression. In this study, a comprehensive analysis was performed across various PCa cohorts to pinpoint lipid metabolism-related genes pivotal in PCa progression. The findings revealed a marked downregulation of STARD4 expression in PCa cell lines. Reduced STARD4 expression was associated with adverse outcomes and specific clinicopathological features. Subsequent in vivo and in vitro studies demonstrated that the overexpression of STARD4 significantly hinders PCa progression.

While STARD4 has been recognized for its role in regulating lipid metabolism in various types of tumours, its specific impact on PCa has remained less clear. In this study, we employed ORO staining alongside measurements of TG and cholesterol levels to investigate the role of STARD4 in lipid metabolism in PCa cells. The results demonstrated that the overexpression of STARD4 significantly reduced lipid accumulation in PCa cells, whereas the knockdown of STARD4 led to increased lipid accumulation. Furthermore, animal experiments were conducted to validate those observations. The results revealed that STARD4 overexpression decreased lipid accumulation in tumour tissues, whereas STARD4 knockdown resulted in increased lipid accumulation. Collectively, these findings indicate the direct involvement of STARD4 in the modulation of intracellular lipid dynamics in PCa. The ER serves as the principal site for cholesterol synthesis and acts as a pivotal cellular organelle in the regulation of cholesterol levels [42]. Within the ER, surplus cholesterol undergoes esterification into cholesterol esters through the catalytic action of acetyl-CoA acetyltransferase, after which the cholesterol esters are stored as LDs [43, 44]. Research has indicated that SREBF2 facilitates the transcriptional activation of STARD4, thereby enhancing STARD4-mediated cholesterol transport, a process deemed critical for SREBF2-induced sorafenib resistance in HCC [22]. Moreover, several investigations have highlighted the role of STARD4 in the modulation of cholesterol levels within the ER and its involvement in ER stress triggered by cholesterol imbalances [45–47]. Given that GSEA revealed a significant correlation between increased STARD4 expression and the activation of the ER stress signalling pathway, subsequent experiments were conducted, the results of which demonstrated that the overexpression of STARD4 markedly elevates the expression of ER stress markers and induces apoptosis in PCa cells.

Functional enrichment analysis revealed a significant correlation between STARD4 and the AR signalling pathway, which is intrinsically linked to the progression of PCa. Consequently, we delved deeper into the relationship between STARD4 and the AR signalling pathway. Given that cholesterol is a precursor in androgen biosynthesis, we initially explored the regulatory role of STARD4 in androgen synthesis. Our findings revealed that the overexpression of STARD4 reduces testosterone levels in PCa cells. Given that androgens bind to and activate AR to exert their physiological effects [48], we investigated whether STARD4 regulates AR expression. Surprisingly, STARD4 overexpression diminishes the stability of the AR protein. Co-IP and GST pull-down experiments confirmed a direct interaction between STARD4 and AR. Subsequent studies revealed that STARD4 promotes the ubiquitination and degradation of the AR protein, thereby modulating the AR signalling pathway. Research has indicated that several ubiquitin E3 ligases, including Skp2, TRAF4, and RNF6, participate in AR protein degradation via the ubiquitin‒proteasome pathway [49–52]. Using the STRING database and the Ubibrowser prediction tool, we identified UBE4B as a potential mediator of AR ubiquitination influenced by STARD4. Further experimentation revealed that STARD4 enhances the interaction between UBE4B and AR, facilitating the ubiquitination and degradation of the AR protein. Additionally, a UBE4B mutant (1140A) failed to restore STARD4-mediated AR protein degradation, underscoring the critical role of UBE4B ubiquitination activity in regulating AR protein stability through STARD4. These findings illuminate the intricate molecular mechanisms governing AR signalling regulation by STARD4.

Enzalutamide, a potent second-generation inhibitor of AR signalling, curtails the proliferation of PCa cells and extends the survival of CRPC patients by impeding the nuclear translocation of activated AR [3, 53]. Regrettably, CRPC eventually develops resistance to enzalutamide due to a combination of mechanisms, such as intratumoural androgen synthesis, the overexpression and gain-of-function mutations of the AR gene, the expression of AR splice variants, the activation of Wnt signalling, and the Warburg effect associated with altered cancer cell metabolism [6, 39, 54]. Using an enzalutamide-resistant cell model (C4-2-EnzR), our investigations revealed that STARD4 overexpression markedly potentiated the inhibitory effects of enzalutamide on PCa cells and reversed enzalutamide resistance. In vivo experiments corroborated these findings, demonstrating that STARD4 overexpression significantly restrains the growth of enzalutamide-resistant tumours. By increasing enzalutamide efficacy through increased apoptosis, diminished proliferation, and reduced lipid droplet size, STARD4 overexpression has emerged as a promising strategy for improving outcomes in patients with advanced or treatment-resistant PCa. This discovery opens new avenues for developing therapeutic approaches focused on modulating STARD4 expression or activity in conjunction with current antiandrogen therapies.

Although we revealed the crucial roles of STARD4 in lipid metabolism regulation and the AR signalling pathway, the intricate molecular regulatory networks underlying these processes remain to be fully elucidated. Further in-depth mechanistic studies are essential to clarify the mechanisms governing UBE4B activity as mediated by STARD4. Additionally, a prospective cohort study is needed to validate the feasibility and efficacy of targeting STARD4 for PCa treatment.

In conclusion, our study offers profound insights into the diverse biological functions of STARD4 in PCa, including its roles in inhibiting tumour growth, regulating lipid metabolism, and modulating the AR signalling pathway. Moreover, STARD4 has the potential to reverse enzalutamide resistance in PCa. These findings not only increase our understanding of the molecular mechanisms underlying PCa progression and drug resistance but also reveal novel therapeutic targets and strategies for combating this formidable disease.

## Supplementary Information

Supplementary Material 1.

## Data Availability

All the data can be obtained by contacting the corresponding author.

## Abbreviations

PCa: Prostate cancer
AR: Androgen receptor
ADT: Androgen deprivation therapy
CRPC: Castration-resistant prostate cancer
STARD4: StAR-related lipid transfer domain protein 4
RT‒qPCR: Real-time quantitative polymerase chain reaction
ORO: Oil Red O
ER: Endoplasmic reticulum
TEM: Transmission electron microscopy
Co-IP: Coimmunoprecipitation
UBE4B: Human ubiquitination factor E4B
ACSL3: Long-chain acyl-CoA synthetase 3
MBOAT2: Membrane-bound O-acyltransferase domain-containing 2
ELOVL5/7: Elongation of very long-chain fatty acids 5/7
SREBP2: Sterol regulatory element-binding protein 2
ATCC: American type culture collection
RPMI: Roswell park memorial institute
K-SMF: Keratinocyte-serum free medium
C4-2-EnzR: C4-2-enzalutamide resistance
STR: Short tandem repeats
FBS: Fetal bovine serum
PI: Propidium iodide
RNA-seq: RNA sequencing
SYBR: Synergy Brands
SDS‒PAGE: Sodium dodecyl sulfate–polyacrylamide gel electrophoresis
PBS: Phosphate-buffered saline
DAPI: 4',6-Diamidino-2-phenylindole
TG: Triglyceride
ELISA: Enzyme-linked immunosorbent assay
TCGA: The cancer genome atlas
GEO: Gene expression omnibus
DKFZ: Dresden and the german cancer research center
MSKCC: Memorial sloan kettering cancer center
GSEA: Gene set enrichment analysis
IHC: Immunohistochemistry
ZO-1: Zonula occludens-1
Bcl-2: B-cell lymphoma 2
EMT: Epithelial‒mesenchymal transition
LD: Lipid droplet
NTD: N-terminal transactivation domain
DBD: DNA-binding domain
LBD: C-terminal ligand-binding domain
CHX: Cycloheximide
MG132: Carbobenzoxyl-L-leucyl-L-leucyl-L-leucine
CQ: Chloroquine
HCC: Hepatocellular carcinoma
Skp2: S-phase kinase-associated protein 2
TRAF4: Tumor necrosis factor receptor-associated factor 4
RNF6: Ring finger protein 6

## References

[1] Siegel RL, Miller KD, Wagle NS, Jemal A. Cancer statistics, 2023. CA Cancer J Clin. 2023;73(1):17–48. 36633525 10.3322/caac.21763

[2] Sekhoacha M, Riet K, Motloung P, Gumenku L, Adegoke A, Mashele S. Prostate cancer review: genetics, diagnosis, treatment options, and alternative approaches. Molecules. 2022;27(17):5730. 36080493 10.3390/molecules27175730 PMC9457814

[3] Wang Y, Chen J, Wu Z, Ding W, Gao S, Gao Y, et al. Mechanisms of enzalutamide resistance in castration-resistant prostate cancer and therapeutic strategies to overcome it. Br J Pharmacol. 2021;178(2):239–61. 33150960 10.1111/bph.15300

[4] Ku SY, Gleave ME, Beltran H. Towards precision oncology in advanced prostate cancer. Nat Rev Urol. 2019;16(11):645–54. 31591549 10.1038/s41585-019-0237-8 PMC6858516

[5] Scher HI, Fizazi K, Saad F, Taplin ME, Sternberg CN, Miller K, et al. Increased survival with enzalutamide in prostate cancer after chemotherapy. N Engl J Med. 2012;367(13):1187–97. 22894553 10.1056/NEJMoa1207506

[6] Buttigliero C, Tucci M, Bertaglia V, Vignani F, Bironzo P, Di Maio M, et al. Understanding and overcoming the mechanisms of primary and acquired resistance to abiraterone and enzalutamide in castration resistant prostate cancer. Cancer Treat Rev. 2015;41(10):884–92. 26342718 10.1016/j.ctrv.2015.08.002

[7] X Y, C C, S C, Z Y, Sp B. Androgen receptor functions in castration-resistant prostate cancer and mechanisms of resistance to new agents targeting the androgen axis. Oncogene. 2014;33(22). Available from: https://pubmed.ncbi.nlm.nih.gov/23752196/. Cited 2024 Dec 18. 10.1038/onc.2013.235 PMC4890635 23752196

[8] Pope ED, Kimbrough EO, Vemireddy LP, Surapaneni PK, Copland JA, Mody K. Aberrant lipid metabolism as a therapeutic target in liver cancer. Expert Opin Ther Targets. 2019;23(6):473–83. 31076001 10.1080/14728222.2019.1615883 PMC6594827

[9] D ZDS, Jc de S, Tm P, B da SM, Rsr J, Sms B, et al. The impact of lipid metabolism on breast cancer: a review about its role in tumorigenesis and immune escape. Cell communication and signaling : CCS. 2023;21(1). Available from: https://pubmed.ncbi.nlm.nih.gov/37370164/. Cited 2024 Dec 18. 10.1186/s12964-023-01178-1 PMC10304265 37370164

[10] M M, R C, F P, G M, N M, M P, et al. Lipid-loaded tumor-associated macrophages sustain tumor growth and invasiveness in prostate cancer. The Journal of experimental medicine. 2022;219(2). Available from: https://pubmed.ncbi.nlm.nih.gov/34919143/. Cited 2024 Dec 18. 10.1084/jem.20210564 PMC8932635 34919143

[11] L Z, C Z, X Y, L L, J H, Y H, et al. Melatonin inhibits lipid accumulation to repress prostate cancer progression by mediating the epigenetic modification of CES1. Clinical and translational medicine. 2021;11(6). Available from: https://pubmed.ncbi.nlm.nih.gov/34185414/. Cited 2024 Dec 18. 10.1002/ctm2.449 PMC8181204 34185414

[12] Zhou L, Song Z, Hu J, Liu L, Hou Y, Zhang X, et al. ACSS3 represses prostate cancer progression through downregulating lipid droplet-associated protein PLIN3. Theranostics. 2021;11(2):841–60. 33391508 10.7150/thno.49384 PMC7738848

[13] Zeković M, Bumbaširević U, Živković M, Pejčić T. Alteration of lipid metabolism in prostate cancer: multifaceted oncologic implications. Int J Mol Sci. 2023;24(2):1391. 36674910 10.3390/ijms24021391 PMC9863986

[14] Han W, Gao S, Barrett D, Ahmed M, Han D, Macoska JA, et al. Reactivation of androgen receptor-regulated lipid biosynthesis drives the progression of castration-resistant prostate cancer. Oncogene. 2018;37(6):710–21. 29059155 10.1038/onc.2017.385 PMC5805650

[15] Siltari A, Syvälä H, Lou YR, Gao Y, Murtola TJ. Role of lipids and lipid metabolism in prostate cancer progression and the tumor’s immune environment. Cancers (Basel). 2022;14(17):4293. 36077824 10.3390/cancers14174293 PMC9454444

[16] Ikonen E, Olkkonen VM. Intracellular Cholesterol Trafficking. Cold Spring Harb Perspect Biol. 2023;15(8):a041404. 37277190 10.1101/cshperspect.a041404 PMC10411867

[17] A EK, W DV, M L, S A, J AM, A K, et al. Macrophage-Derived Cholesterol Contributes to Therapeutic Resistance in Prostate Cancer. Cancer research. 2021;81(21). Available from: https://pubmed.ncbi.nlm.nih.gov/34301759/. Cited 2024 Dec 18. 10.1158/0008-5472.CAN-20-4028 PMC8563406 34301759

[18] Y Y, Y B, Y H, Y Z, J C, L M, et al. PTEN Loss Promotes Intratumoral Androgen Synthesis and Tumor Microenvironment Remodeling via Aberrant Activation of RUNX2 in Castration-Resistant Prostate Cancer. Clinical cancer research: an official journal of the American Association for Cancer Research. 2018;24(4). Available from: https://pubmed.ncbi.nlm.nih.gov/29167276/. Cited 2024 Dec 18. 10.1158/1078-0432.CCR-17-2006 PMC5816982 29167276

[19] Cai C, Chen S, Ng P, Bubley GJ, Nelson PS, Mostaghel EA, et al. Intratumoral de novo steroid synthesis activates androgen receptor in castration-resistant prostate cancer and is upregulated by treatment with CYP17A1 inhibitors. Cancer Res. 2011;71(20):6503–13. 21868758 10.1158/0008-5472.CAN-11-0532 PMC3209585

[20] Clark BJ. The mammalian START domain protein family in lipid transport in health and disease. J Endocrinol. 2012;212(3):257–75. 21965545 10.1530/JOE-11-0313

[21] Alpy F, Tomasetto C. START ships lipids across interorganelle space. Biochimie. 2014;96:85–95. 24076129 10.1016/j.biochi.2013.09.015

[22] Yue X, Kong Y, Zhang Y, Sun M, Liu S, Wu Z, et al. SREBF2-STARD4 axis confers sorafenib resistance in hepatocellular carcinoma by regulating mitochondrial cholesterol homeostasis. Cancer Sci. 2023;114(2):477–89. 35642354 10.1111/cas.15449 PMC9899602

[23] Li G, Wu T, Li H, Wei C, Sun Y, Gao P, et al. Construction of a tumor mutational burden-derived LncRNA prognostic computational framework associated with therapy sensitivity in skin cutaneous melanoma. J Transl Med. 2024;22(1):966. 39449143 10.1186/s12967-024-05732-4 PMC11515383

[24] Tan M, Lin X, Chen H, Ye W, Yi J, Li C, et al. Sterol regulatory element binding transcription factor 1 promotes proliferation and migration in head and neck squamous cell carcinoma. PeerJ. 2023;11:e15203. 37090107 10.7717/peerj.15203 PMC10117388

[25] Zhang M, Xiang Z, Wang F, Shan R, Li L, Chen J, et al. Stard4 promotes breast cancer cell malignancy. Oncol Rep. 2020;44(6):2487–502. 33125124 10.3892/or.2020.7802 PMC7610339

[26] Li Q, Zeng K, Chen Q, Han C, Wang X, Li B, et al. Atractylenolide I inhibits angiogenesis and reverses sunitinib resistance in clear cell renal cell carcinoma through ATP6V0D2-mediated autophagic degradation of EPAS1/HIF2α. Autophagy. 2025;21:619–38. 10.1080/15548627.2024.2421699 PMC11849937 39477683

[27] Hetz C. The unfolded protein response: controlling cell fate decisions under ER stress and beyond. Nat Rev Mol Cell Biol. 2012;13(2):89–102. 22251901 10.1038/nrm3270

[28] Sano R, Reed JC. ER stress-induced cell death mechanisms. Biochim Biophys Acta. 2013;1833(12):3460–70. 23850759 10.1016/j.bbamcr.2013.06.028 PMC3834229

[29] Culig Z, Santer FR. Androgen receptor signaling in prostate cancer. Cancer Metastasis Rev. 2014;33(2–3):413–27. 24384911 10.1007/s10555-013-9474-0

[30] Zhu S, Zhao D, Yan L, Jiang W, Kim JS, Gu B, et al. BMI1 regulates androgen receptor in prostate cancer independently of the polycomb repressive complex 1. Nat Commun. 2018;9(1):500. 29402932 10.1038/s41467-018-02863-3 PMC5799368

[31] Antoniou N, Lagopati N, Balourdas DI, Nikolaou M, Papalampros A, Vasileiou PVS, et al. The Role of E3, E4 Ubiquitin Ligase (UBE4B) in Human Pathologies. Cancers (Basel). 2019;12(1):62. 31878315 10.3390/cancers12010062 PMC7017255

[32] Shao X, Zhu J, Shi Y, Fang H, Chen J, Zhang Y, et al. Upregulated UBE4B expression correlates with poor prognosis and tumor immune infiltration in hepatocellular carcinoma. Aging (Albany NY). 2022;14(23):9632–46. 36470669 10.18632/aging.204414 PMC9792214

[33] Wu S, Xie L, Cheng S, Fan Z, Sang H, Li Q. Ube4b promotes the development of lung adenocarcinoma by enhancing proliferation, migration and glycolysis via PP2A/AKT signaling. Pathol Res Pract. 2022;232:153762. 35220170 10.1016/j.prp.2022.153762

[34] Weng C, Chen Y, Wu Y, Liu X, Mao H, Fang X, et al. Silencing UBE4B induces nasopharyngeal carcinoma apoptosis through the activation of caspase3 and p53. Onco Targets Ther. 2019;12:2553–61. 31040698 10.2147/OTT.S196132 PMC6459139

[35] Wang B, Wu H, Chai C, Lewis J, Pichiorri F, Eisenstat DD, et al. Microrna-1301 suppresses tumor cell migration and invasion by targeting the p53/UBE4B pathway in multiple human cancer cells. Cancer Lett. 2017;401:20–32. 28483517 10.1016/j.canlet.2017.04.038

[36] Mohanty S, Han T, Choi YB, Lavorgna A, Zhang J, Harhaj EW. The E3/E4 ubiquitin conjugation factor UBE4B interacts with and ubiquitinates the HTLV-1 Tax oncoprotein to promote NF-κB activation. PLoS Pathog. 2020;16(12):e1008504. 33362245 10.1371/journal.ppat.1008504 PMC7790423

[37] Wang T, Song W, Chen Y, Chen R, Liu Z, Wu L, et al. Flightless i homolog represses prostate cancer progression through targeting androgen receptor signaling. Clin Cancer Res. 2016;22(6):1531–44. 26527749 10.1158/1078-0432.CCR-15-1632

[38] Desai K, McManus JM, Sharifi N. Hormonal therapy for prostate cancer. Endocr Rev. 2021;42(3):354–73. 33480983 10.1210/endrev/bnab002 PMC8152444

[39] Handle F, Prekovic S, Helsen C, Van den Broeck T, Smeets E, Moris L, et al. Drivers of AR indifferent anti-androgen resistance in prostate cancer cells. Sci Rep. 2019;9:13786. 31551480 10.1038/s41598-019-50220-1 PMC6760229

[40] Salji MJ, Blomme A, Däbritz JHM, Repiscak P, Lilla S, Patel R, et al. Multi-omics & pathway analysis identify potential roles for tumor N-acetyl aspartate accumulation in murine models of castration-resistant prostate cancer. iScience. 2022;25(4):104056. 35345457 10.1016/j.isci.2022.104056 PMC8957019

[41] Stopsack KH, Gerke TA, Andrén O, Andersson SO, Giovannucci EL, Mucci LA, et al. Cholesterol uptake and regulation in high-grade and lethal prostate cancers. Carcinogenesis. 2017;38(8):806–11. 28595267 10.1093/carcin/bgx058 PMC6074871

[42] Mesmin B, Pipalia NH, Lund FW, Ramlall TF, Sokolov A, Eliezer D, et al. Stard4 abundance regulates sterol transport and sensing. Mol Biol Cell. 2011;22(21):4004–15. 21900492 10.1091/mbc.E11-04-0372 PMC3204063

[43] Olzmann JA, Carvalho P. Dynamics and functions of lipid droplets. Nat Rev Mol Cell Biol. 2019;20(3):137–55. 30523332 10.1038/s41580-018-0085-z PMC6746329

[44] Qiu B, Ackerman D, Sanchez DJ, Li B, Ochocki JD, Grazioli A, et al. HIF2α-dependent lipid storage promotes endoplasmic reticulum homeostasis in clear-cell renal cell carcinoma. Cancer Discov. 2015;5(6):652–67. 25829424 10.1158/2159-8290.CD-14-1507 PMC4456212

[45] Soffientini U, Graham A. Intracellular cholesterol transport proteins: roles in health and disease. Clin Sci Lond. 2016;130(21):1843–59. 27660308 10.1042/CS20160339

[46] Yamada S, Yamaguchi T, Hosoda A, Iwawaki T, Kohno K. Regulation of human STARD4 gene expression under endoplasmic reticulum stress. Biochem Biophys Res Commun. 2006;343(4):1079–85. 16579971 10.1016/j.bbrc.2006.03.051

[47] Rodriguez-Agudo D, Calderon-Dominguez M, Ren S, Marques D, Redford K, Medina-Torres MA, et al. Subcellular localization and regulation of StarD4 protein in macrophages and fibroblasts. Biochim Biophys Acta. 2011;1811(10):597–606. 21767660 10.1016/j.bbalip.2011.06.028 PMC3156897

[48] Hou Z, Huang S, Li Z. Androgens in prostate cancer: a tale that never ends. Cancer Lett. 2021;516:1–12. 34052327 10.1016/j.canlet.2021.04.010

[49] Singh R, Meng H, Shen T, Lumahan LEV, Nguyen S, Shen H, et al. TRAF4-mediated nonproteolytic ubiquitination of androgen receptor promotes castration-resistant prostate cancer. Proc Natl Acad Sci U S A. 2023;120(20):e2218229120. 37155905 10.1073/pnas.2218229120 PMC10193960

[50] Xu K, Shimelis H, Linn DE, Jiang R, Yang X, Sun F, et al. Regulation of androgen receptor transcriptional activity and specificity by RNF6-induced ubiquitination. Cancer Cell. 2009;15(4):270–82. 19345326 10.1016/j.ccr.2009.02.021 PMC2848969

[51] Li B, Lu W, Yang Q, Yu X, Matusik RJ, Chen Z. Skp2 regulates androgen receptor through ubiquitin-mediated degradation independent of Akt/mTOR pathways in prostate cancer. Prostate. 2014;74(4):421–32. 24347472 10.1002/pros.22763 PMC4062570

[52] Linn DE, Yang X, Xie Y, Alfano A, Deshmukh D, Wang X, et al. Differential regulation of androgen receptor by PIM-1 kinases via phosphorylation-dependent recruitment of distinct ubiquitin E3 ligases. J Biol Chem. 2012;287(27):22959–68. 22584579 10.1074/jbc.M111.338350 PMC3391098

[53] Miller KD, Nogueira L, Devasia T, Mariotto AB, Yabroff KR, Jemal A, et al. Cancer treatment and survivorship statistics, 2022. CA Cancer J Clin. 2022;72(5):409–36. 35736631 10.3322/caac.21731

[54] Yuan F, Hankey W, Wu D, Wang H, Somarelli J, Armstrong AJ, et al. Molecular determinants for enzalutamide-induced transcription in prostate cancer. Nucleic Acids Res. 2019;47(19):10104–14. 31501863 10.1093/nar/gkz790 PMC6821169

