# Supplementary material for: STARD4 suppresses tumorigenesis and attenuates enzalutamide resistance via lipid metabolic reprogramming and AR stabilization in prostate cancer
Source: J Exp Clin Cancer Res. 2025 Dec 2;44:318. doi: 10.1186/s13046-025-03600-7 (PMC12699821; doi:10.1186/s13046-025-03600-7)
Supplement: Supplementary file 1 — Supplementary Material 1. [file 13046_2025_3600_MOESM1_ESM.pdf]

STARD4 Suppresses Tumorigenesis and Attenuates Enzalutamide Resistance via Lipid Metabolic Reprogramming and AR Stabilization in Prostate Cancer

Yi Zhang[1, †], Xi Wang[2, †], Jiuyi Wang[1, †], Ke Ma[1, †], Lei Jia[1], Bo Liu[2], Xianglin Yuan[2], Qiang Li[1, *], Qinzhang Wang[1, *], Qinyu Li[2, *], Kai Zeng[1, *]

[1]Department of Urology, the First Affiliated Hospital of Shihezi University, Shihezi, Xinjiang, China
[2]Department of Oncology, Tongji Hospital, Tongji Medical College, Huazhong University of Science and Technology, Wuhan, Hubei, China

† These authors contributed to this work equally and shared first authorship.

Corresponding to:

Kai Zeng, zengkai@shzu.edu.cn

Qinyu Li, qinyuli2022tjh@163.com

Qinzhang Wang, wqz1969@sina.com

Qiang Li, liqiangbl123@sina.com

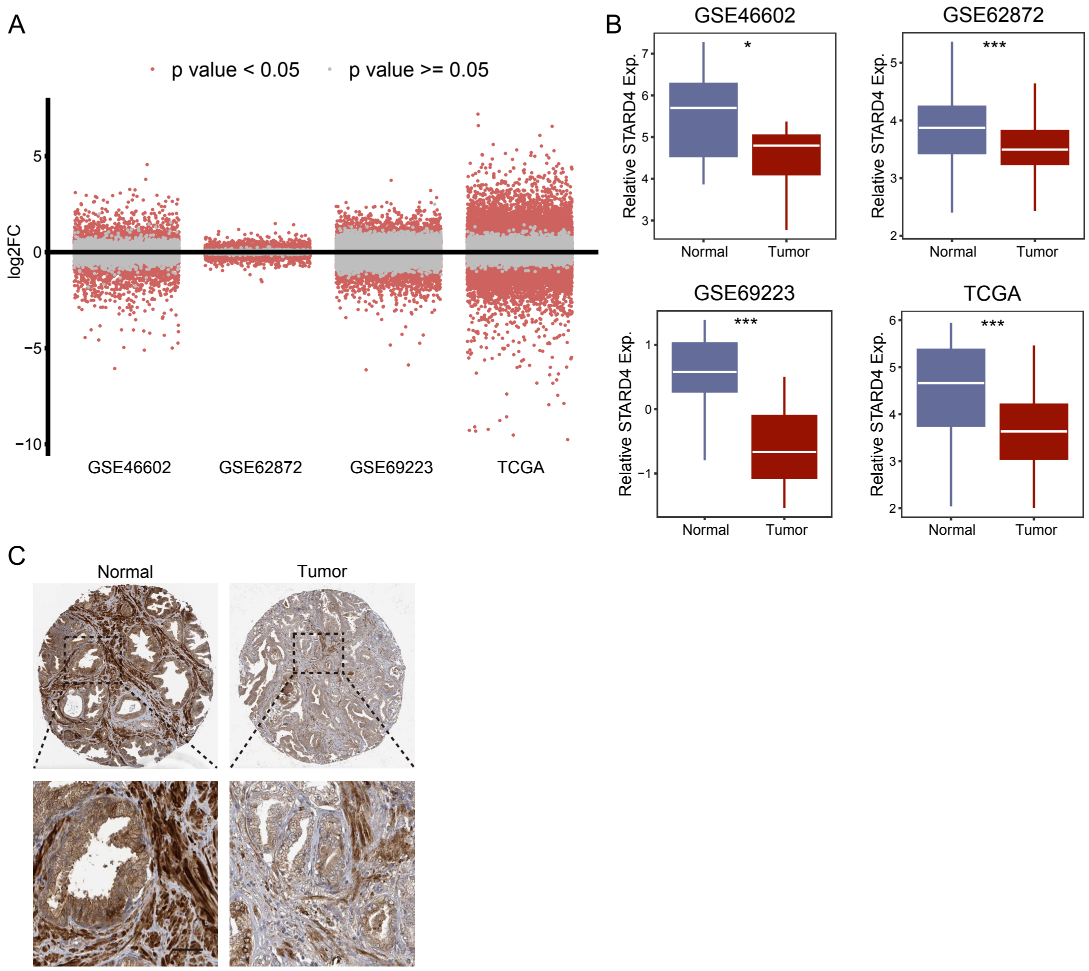

Figure S1. A The volcano map illustrates the differentially expressed genes between normal prostate and PCa samples from the TCGA PCa database and three different PCa transcriptome datasets (GSE62872, GSE46602, and GSE69223). **B** Comparative STARD4 expression in normal prostate versus PCa tumors across the indicated datasets. **C** IHC staining of STARD4 expression in PCa and normal prostate tissues from the HPA database. Scale bar: 200 μm. Unpaired two-tailed Student's t test (**B**). (*, $p < 0.05$; ***, $p < 0.001$). Data are presented as the mean ± SD.

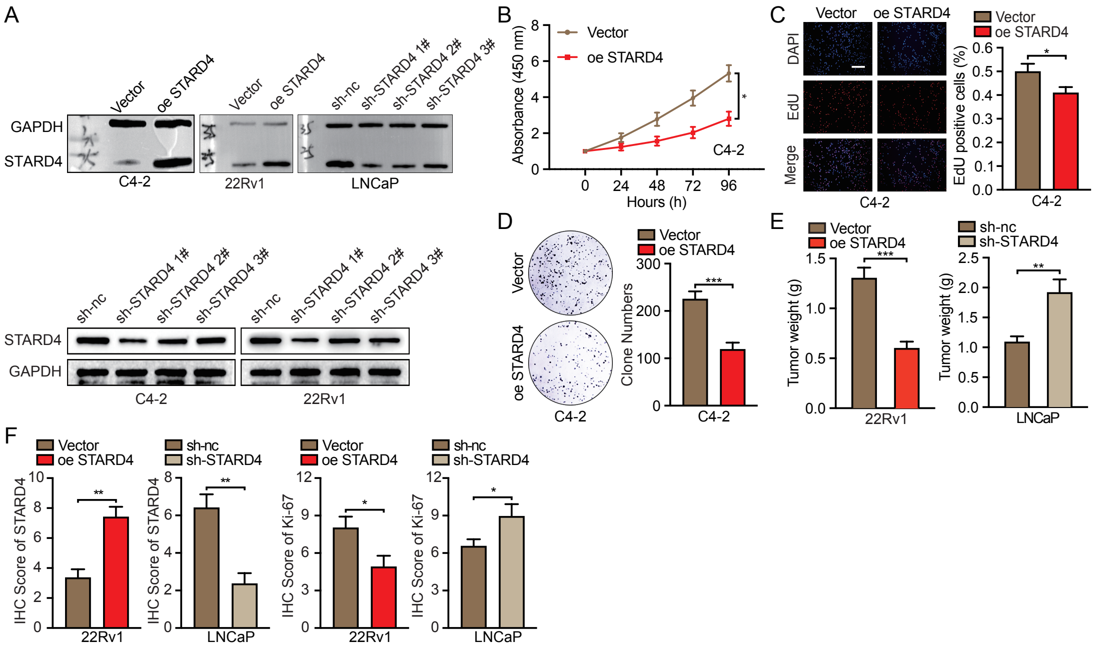

Figure S2. **A** Western blotting was used to determine STARD4 protein overexpression in C4-2 and 22Rv1 cells, and STARD4 knockdown in LNCaP, C4-2, and 22Rv1 cells. **B** Cell viability analysis using the CCK-8 assay in the C4-2 cell line with STARD4 overexpression ($n = 3$). **C-D** Evaluation of cell proliferation capacity using the EdU and Colony formation assay in C4-2 cells with stable STARD4 overexpression ($n = 3$). Scale bar: 50 μm. **E** Tumor tissues from the indicated treatment groups were weighed and shown in the histograms ($n = 5$). **F** Quantification of STARD4 and Ki-67 levels according to IHC scores ($n = 5$). Unpaired two-tailed Student's t test (**C, D, E left, F**); Mann-Whitney U test (**E right**); Two-way ANOVA (**B**). (*, $p < 0.05$; **, $p < 0.01$; ***, $p < 0.001$). Data are presented as the mean ± SD.

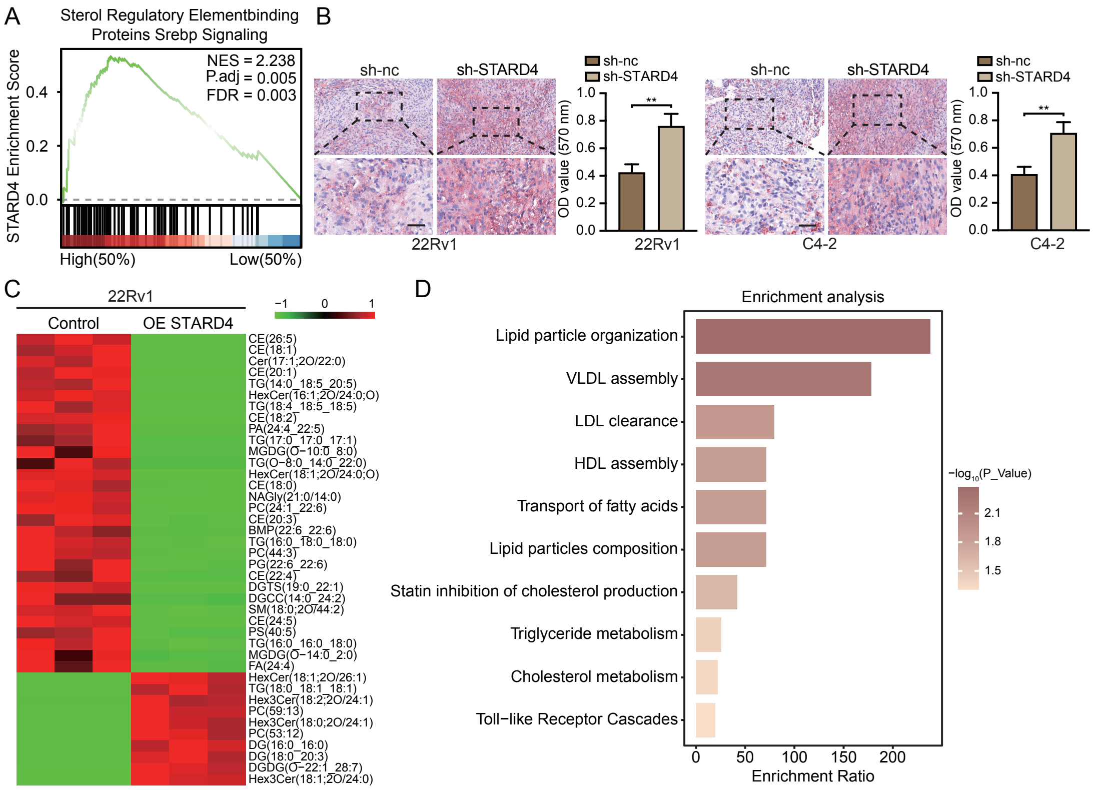

Figure S3. A GSEA revealed a close association of sterol regulatory element binding proteins srebp signaling with STARD4 high expression. **B** OrO staining of xenograft tumor sections between the sh-nc and sh-STARD4 groups in 22Rv1 and C4-2 cells (*n* = 5). Scale bar: 50 µm. **C** LC-MS-based untargeted lipidomic profiling of intracellular lipids in 22Rv1 cells with or without stable overexpression of STARD4 (*n*= 3). **D** KEGG pathway enrichment analysis based on the LC-MS-based lipidomic analysis results. Unpaired two-tailed Student's t test (**B**). (**, $p < 0.01$). Data are presented as the mean ± SD.

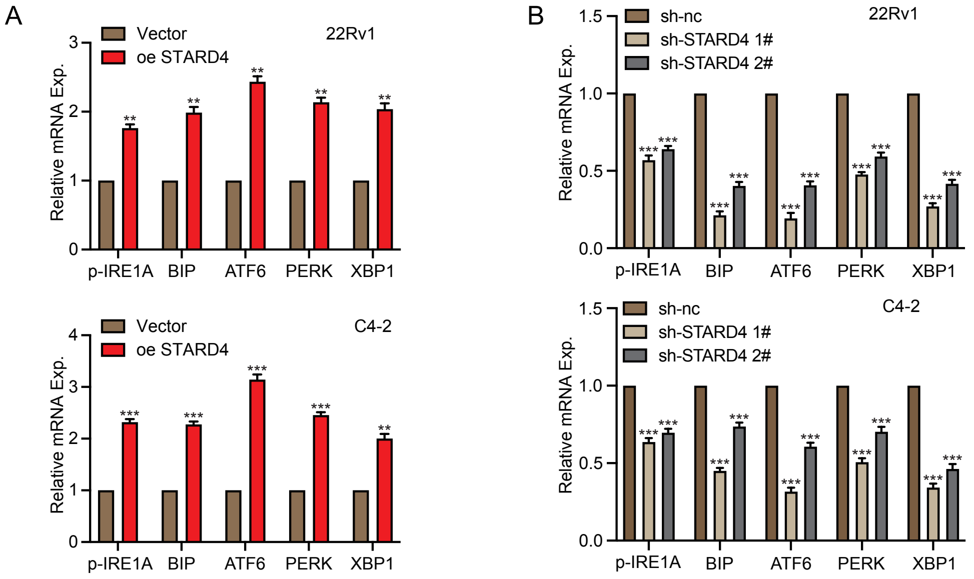

Figure S4. A-B The mRNA levels of ER stress markers (p-IRE1A, BIP, ATF6, PERK, and XBP1) in 22Rv1 and C4-2 cells stably overexpressing or knocking down STARD4 were measured by RT-qPCR (n = 3). Unpaired two-tailed Student's t test (**A**); One-way ANOVA (**B**). (**, $p < 0.01$; ***, $p < 0.001$). Data are presented as the mean ± SD.

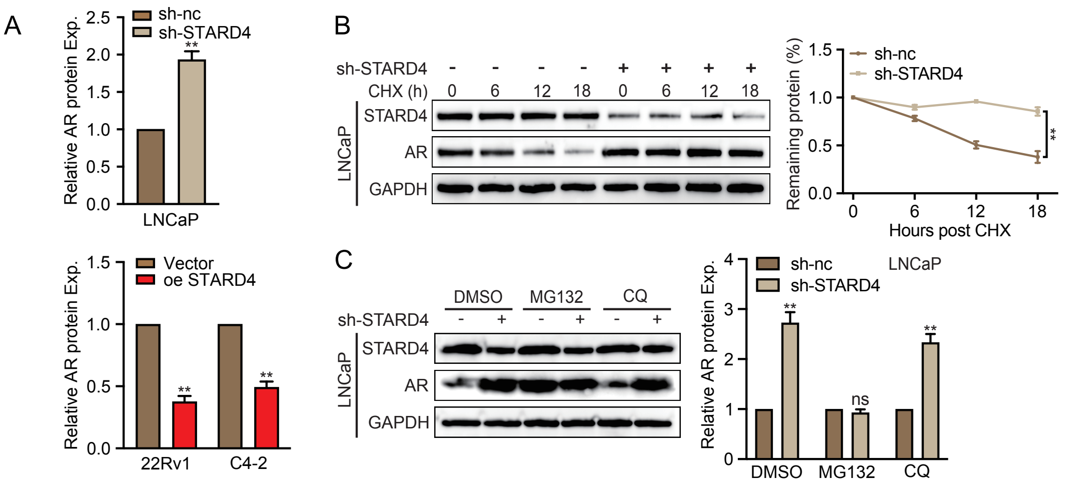

Figure S5. A AR protein levels in the indicated groups are shown in histograms (n = 3). **B** Protein synthesis inhibition in LNCaP cells with STARD4 knockdown using CHX (10 μM), with AR protein levels determined by western blotting at 0, 6, 12, and 18 h (n = 3). **C** Treatment of 22Rv1 and C4-2 cells stably knocking down STARD4 with DMSO, chloroquine (CQ, 20 μM), or MG132 (50 μM), and analysis of AR protein levels by western blotting (n = 3). Unpaired two-tailed Student's t test (**A, C**); Two-way ANOVA (**B**). (ns, no significance; **, p < 0.01). Data are presented as the mean ± SD.

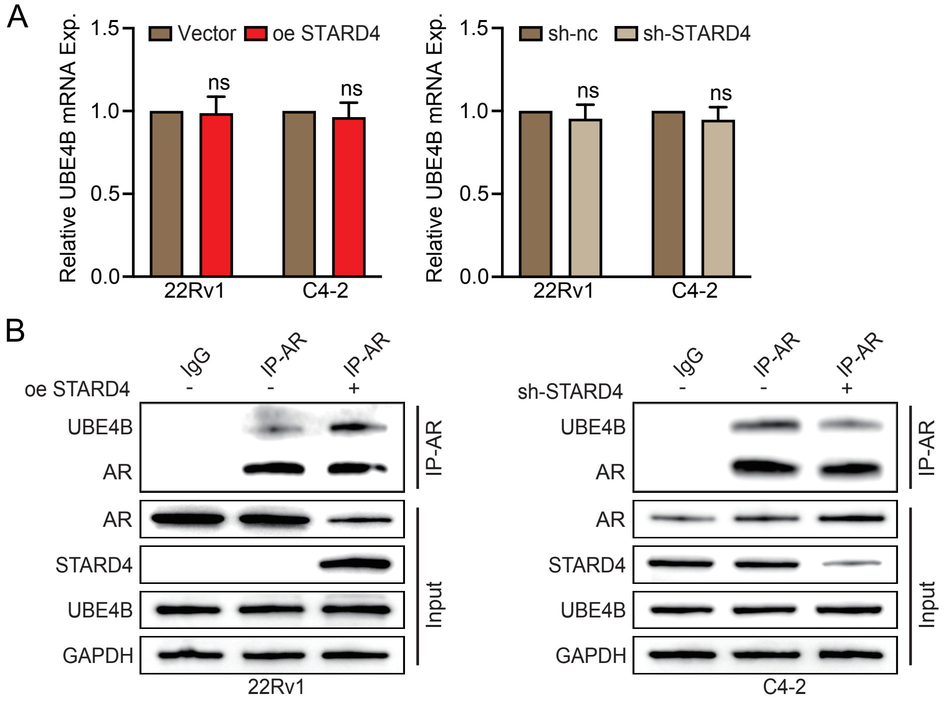

Figure S6. A The levels of UBE4B mRNA in 22Rv1 and C4-2 cells with STARD4 overexpression or knockdown were measured relative to those in control cells (n = 3). **B** 22Rv1 cells were transfected with a STARD4 overexpression plasmid, while C4-2 cells were transfected with a STARD4 knockdown plasmid. Co-IP with IgG or AR antibody was followed by western blotting with the indicated antibodies. Unpaired two-tailed Student's t test (**A**). (ns, no significance). Data are presented as the mean ± SD.

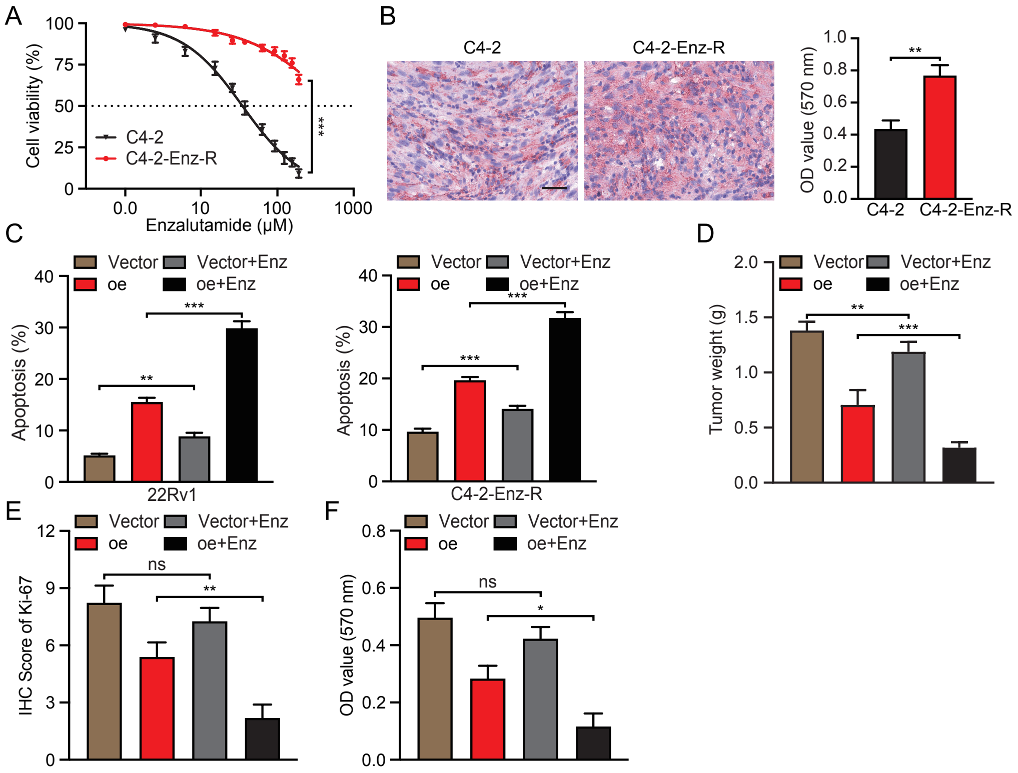

Figure S7. A C4-2 and C4-2-EnzR cells were treated with different concentrations of enzalutamide for 48 h, and cell viability was measured by CCK-8 assay ($n = 5$). **B** Lipid deposition in wildtype or enzalutamide-resistant xenograft tumor sections by OrO staining ($n = 5$). Scale bar: 50 μm. **C** The histograms represent the average percentage of apoptosis in the indicated groups treated with DMSO or enzalutamide ($n = 3$). **D** The histogram shows the mean tumor weights after harvesting the xenograft tumors from the indicated groups ($n = 5$). **E** The left graph shows the quantification of Ki-67 levels from the indicated groups according to IHC scores ($n = 5$). **F** Quantification of relative OrO-stained signals from the indicated groups is shown in the graph at right ($n = 5$). Unpaired two-tailed Student's t test (**B, C, D, E, F**). (ns, no significance; *, $p < 0.05$; **, $p < 0.01$; ***, $p < 0.001$). Data are presented as the mean ± SD.

Supplementary Table 1. Sequence of primers for qRT-PCR analysis

**Primers used in q-PCR analysis**		
GAPDH	Forward primer	CGCTCTCTGCTCCTCCTGTTC
	Reverse primer	ATCCGTTGACTCCGACCTTCAC
STARD4	Forward primer	CTCTACAAAGCCCAAGGTG
	Reverse primer	TCATCAAGCTGTCCCAATC
UBE4B	Forward primer	GAGAAAAGCGGAGCCTCAGT
	Reverse primer	GGTCCTTCCAAGAGACACGG
AR	Forward primer	AAGCAGGGATGACTCTGGGA
	Reverse primer	CTGGGTTGTCTCCTCAGTGG
IRE1A	Forward primer	CACAGTGACGCTTCCTGAAAC
	Reverse primer	GCCATCATTAGGATCTGGGAGA
BIP	Forward primer	GAAAGAAGGTTACCCATGCAGT
	Reverse primer	CAGGCCATAAGCAATAGCAGC
ATF6	Forward primer	TCCTCGGTCAGTGGACTCTTA
	Reverse primer	CTTGGGCTGAATTGAAGGTTTTG
PERK	Forward primer	GGAAACGAGAGCCGGATTTATT
	Reverse primer	ACTATGTCCATTATGGCAGCTTC
XBP1	Forward primer	CCCTCCAGAACATCTCCCCAT
	Reverse primer	ACATGACTGGGTCCAAGTTGT

Supplementary Table 2. Primary antibodies used in the study

Antibody	Company (Cat. No.)	Working dilutions
STARD4	Abcam (ab202060)	WB:1/1000, IF:1/1000
STARD4	Thermo (PA5-67611)	IHC:1/100
AR	Proteintech (66747-1-IG)	WB:1/1000, IF:1/100
ZO-1	Abcam (ab190085)	WB:1/1000
N-cadherin	Abcam (ab98952)	WB:1/1000
Vimentin	Abcam (ab8978)	WB:1/1000
BCL-2	Abcam (ab32124)	WB:1/1000
BAX	Abcam (ab32503)	WB:1/5000
Caspase3	Abcam (ab32351)	WB:1/5000
c-caspase3	Abcam (ab32042)	WB:1/500
IRE1A-alpha(p-Ser724)	NOVUS (NB100-2323)	WB:1/1000
BIP	CST (3177)	WB:1/1000
ATF6	CST (65880S)	WB:1/1000
PERK	CST (3192S)	WB:1/1000
XBP1	Abcam (ab37152)	WB:1/1000
UBE4B	Abcam (ab126759)	WB:1/1000
FANCG	Abcam (ab151516)	WB:1/1000
TRIM25	Abcam (ab167154)	WB:1/2000
MDM2	CST (86934)	WB:1/1000
STUB1	CST (C3B6)	WB:1/1000
Ubiquitin	Abcam (ab7254)	WB:1/1000
Ki-67	Abcam (ab16667)	IHC:1/200
Mouse anti Flag	Abclone (AE005)	WB:1/2000
Rabbit anti Flag	Abclone (AE063)	WB:1/2000
Rabbit anti GST	Abclone (AE006)	WB:1/1500
Rabbit anti HA	Abclone (AE105)	WB:1/2000
GAPDH	Proteintech (60004-1-Ig)	WB:1/5000
**Secondary Antibody**		
Goat Anti-Rabbit	Abclone (AS014)	WB:1/5000
Goat Anti-Mouse	Abclone (AS003)	WB:1/5000
Cy3 Goat Anti-Rabbit	Servicebio (GB21303)	IF:1/500
Cy3 Donkey Anti-Mouse	Servicebio (GB21401)	IF:1/500
FITC Goat Anti-Mouse	Servicebio (GB22301)	IF:1/500
FITC Goat Anti-Rabbit	Servicebio (GB22303)	IF:1/500
